# Horizontal transfer of a non-autonomous *Helitron* among insect and viral genomes

**DOI:** 10.1186/s12864-015-1318-6

**Published:** 2015-02-27

**Authors:** Brad S Coates

**Affiliations:** United States Department of Agriculture, Agricultural Research Service, Corn Insects & Crop Genetics Research Unit, Iowa State University, Ames, IA 50011 USA; Department of Entomology, Iowa State University, Ames, IA 50011 USA

**Keywords:** Horizontal transposon transfer, Viral vectors

## Abstract

**Background:**

The movement of mobile elements among species by horizontal transposon transfer (HTT) influences the evolution of genomes through the modification of structure and function. *Helitrons* are a relatively new lineage of DNA-based (class II) transposable elements (TEs) that propagate by rolling-circle replication, and are capable of acquiring host DNA. The rapid spread of *Helitrons* among animal lineages by HTT is facilitated by shuttling in viral particles or by unknown mechanisms mediated by close organism associations (e.g. between hosts and parasites).

**Results:**

A non-autonomous *Helitron* independently annotated as BmHel-2 from *Bombyx mori* and the MITE01 element from *Ostrinia nubilalis* was predicted in the genomes of 24 species in the insect Order Lepidoptera. Integrated *Helitrons* retained ≥ 65% sequence identity over a 250 bp consensus, and were predicted to retain secondary structures inclusive of a 3′-hairpin and a 5′-subterminal inverted repeat. Highly similar Hel-2 copies were predicted in the genomes of insects and associated viruses, which along with a previous documented case of real-time virus-insect cell line transposition suggests that this *Helitron* has likely propagated by HTT.

**Conclusions:**

These findings provide evidence that insect virus may mediate the HTT of *Helitron*-like TEs. This movement may facilitate the shuttling of DNA elements among insect genomes. Further sampling is required to determine the putative role of HTT in insect genome evolution.

**Electronic supplementary material:**

The online version of this article (doi:10.1186/s12864-015-1318-6) contains supplementary material, which is available to authorized users.

## Background

*Helitrons* are class II DNA transposable elements (TEs) that were initially discovered in the *Arabidopsis thaliana* genome and have subsequently been described in a number of diverse eukaryotic genomes where they occupy 1.3 to 6.6% of the total nucleic acid content [1-5]. In contrast to the cut-and-paste propagation of other class II TEs, *Helitrons* transpose in a conservative fashion where original integrations are retained due to the synthesis of daughter TEs by rolling circle replication (RCR) [1]. This type of replication is similar to that used by ssDNA viruses, bacteria, and plasmids, and involves the enzymatic activity of Replicase/Helicase (RepHel) proteins. Eukaryotic RepHels show strong homology with Rep domain-containing proteins encoded by ssDNA geminiviruses such that they are presumed to have been acquired by HTT [6], and mediate RCR of autonomous as well as non-autonomous *Helitron* elements in the same genome. Nascent *Helitron* copies integrate at novel genome locations between AT or TT di-nucleotides and do not produce target site duplications. The integration of *Helitrons* in plant genomes are believed to involve the transposase, RPA-TPase, which shares homology to the largest subunit of the replication protein A (RPA70). Accumulating evidence indicates that *Helitron* integrations can have functional consequences resulting from gene knockouts that lead to observable phenotypic variants [7,8], or insertion mutations that change the genetic architecture and the expression of affected genes [9]. Moreover, *Helitrons* have been implicated in exon shuffling [10], transcription read-through [11], and the mobilization of acquired host genome regions [5,12,13].

*Helitron* integrations in plant genomes can be highly cryptic and difficult to predict outside of conserved 5′-TC and 3′-CTRR termini, and a stem-loop structure near the 3′-terminus [12,14]. In many instances, the comparison of haplotype variants in a species or sophisticated computer algorithms are relied upon to predict the myriad of variant *Helitron* integrations in or among genomes. In contrast, *Helitron* sequences show high levels of intraspecific conservation among integrations in *Drosophila* species [4], the little brown bat, *Myotis lucifugus* [15] and among multiple species in the insect Order Lepidoptera [5,13,16,17]. Additionally, conserved secondary structures are predicted among *Helitrons* from Lepidoptera and Diptera, which form two inverted repeats [a 5′-inverted repeat (5′-IR), and base pairing between subterminal inverted repeats (SIRs)] that may be required for replication, mobilization, or integration [4,13,18]. This dramatic sequence and structural conservation among *Helitrons* in the genomes of divergent insect species was surmised by some to be a consequence of a rapid burst in transposition within and among genomes that has been facilitated by HTT. Specifically, horizontal transfer broadly refers to the exchange of genetic material between established lineages and has been detected for transposons, group I introns, and entire gene sequences [18,19], yet details on the exact mechanism of HTT remain somewhat elusive. Endoparasitic and endosymbiotic organisms can acquire segments of host DNA by intermolecular recombination [20] or following the integration of host-derived mobile genetic elements [21]. The infection of multiple host species by these pathogens has been implicated as a mechanism that vectors DNA across species boundaries. In a recent study, the rapid proliferation of two *Helitrons*, *Heligloria* and *Helisimi*, among insect species were predicted to involve HTT mediated by the dsDNA *Cotesia plutella* and *C. sesamiae* bracoviruses [17]. The *C. plutella* and *C. sesamiae* bracoviruses are aptly named due to the respective infection of the hymenopteran wasps *C. plutella* and *C. sesamiae* that are in-turn ectoparasitoids of several stem boring larval Lepidoptera including *Plutella xylostella* [22], *Sesamia calamistis*, *Busseola fusca*, *Chilo partellus*, and *Chilo orichalcociliellus* [23]. A *Helitron* with high shared sequence identity in the genome of a virus and insect host species provided comparative evidence that these mobile DNA elements are moving between genomes, and that HTT is likely a mechanism by which *Helitrons* have colonized the genomes of related insects [17]. Furthermore, this evidence agreed with prior finding that TEs can be shuttled among genomes by virus- and parasite-host insect interactions [24,25].

Despite this evidence, the evolutionary scope or the methods by which HTT occur remains unclear. For instance, the HTT of DNA transposons between the genomes of animal hosts and their parasitic insects was recently detected, where it was hypothesized that intracellular parasites, viruses, or extrachromosomal material might vector TE movements between genomes of different species, such as between the blood-feeding hexapod *Rhodnius prolixus* and tetrapods [26]. This evidence suggested that HTT of genetic material even among highly divergent species may be more prevalent than previously envisioned. Research herein provides evidence that a conserved non-autonomous *Helitron* has recently invaded the genomes of multiple herbivorous insect species, and may have resulted from HTT vectored by viral particles. Re-annotation of the spontaneous vsk-1d1 *Autographa californica* nucleopolyhedrovirus insertion element [27,28] as a non-autonomous *Helitron* with high similarity to TEs in *Trichoplusia ni* and *Spodoptera frugiperda* cell lines provided putative evidence for contemporary HTT of this insect-derived *Helitron*. These data suggest that *Helitrons* may be capable of crossing taxonomic boundaries and that HTT might be a potent force in shaping genome evolution.

## Results and discussion

### Prediction and annotation of highly conserved *Helitron* integrations

The transposition of novel mobile genetic elements into a genome affects not only the genetic structure and function, but can influence the evolutionary trajectories of a species. High degrees of DNA sequence similarity among copies of a TE in a genome are considered to be evidence of a recent invasion, wherein rapid transposition has not been subject to host repression and substitution mutations have not accumulated in the “young” propagates [29,30]. Additionally, evidence including unanticipated high sequence identity in TE families across the genomes of many taxa has been used to support the notion that mobile elements move across species boundaries [17,26], where horizontal transposon transfer (HTT) is increasingly being viewed as a potent mechanism that influences genome evolution [31]. Indeed, the alignment of two previously annotated TEs, a miniature inverted repeat transposable element from *Ostrinia nubilalis*, OnMITE01 [32], and a *Helitron* element from *B. mori*, BmHel-2 [5], generated a 339 bp consensus that showed 66.2% sequence similarity and 77.55% similarity between 50 bp at the 5′-end (Additional file 1) despite these non-protein coding sequences being from species that have estimated divergence times of > 100 mya [33]. Results from RepBase searches also indicated that a 149 bp region of OnMITE01 (positions 10 to 159) showed 79.2% similarity (hit score = 619) to nucleotides 973 to 827 of the 984 bp *Heliconius melpomene* element *Helitron*-like-4a_Hmel [34]. This was not unanticipated since this *Helitron* was previously shown to share sequence similarity across lepidopteran genomes for elements in the assembled genomes of *B. mori*, *Danus plexippus*, *H. melpomene*, and *Manduca sexta* [5], and also to retain ≥ 74.4% homology between the genomes of *O. nubilalis* and multiple lepidopteran species including *B. mori* [32]. Due to the predicted co-ancestry of BmHel-2 and OnMITE01, and to adapt a common nomenclature for orthologous copies of the same element, the OnMITE01 element is from hereon referred to as OnHel-2.

**Figure 1:**
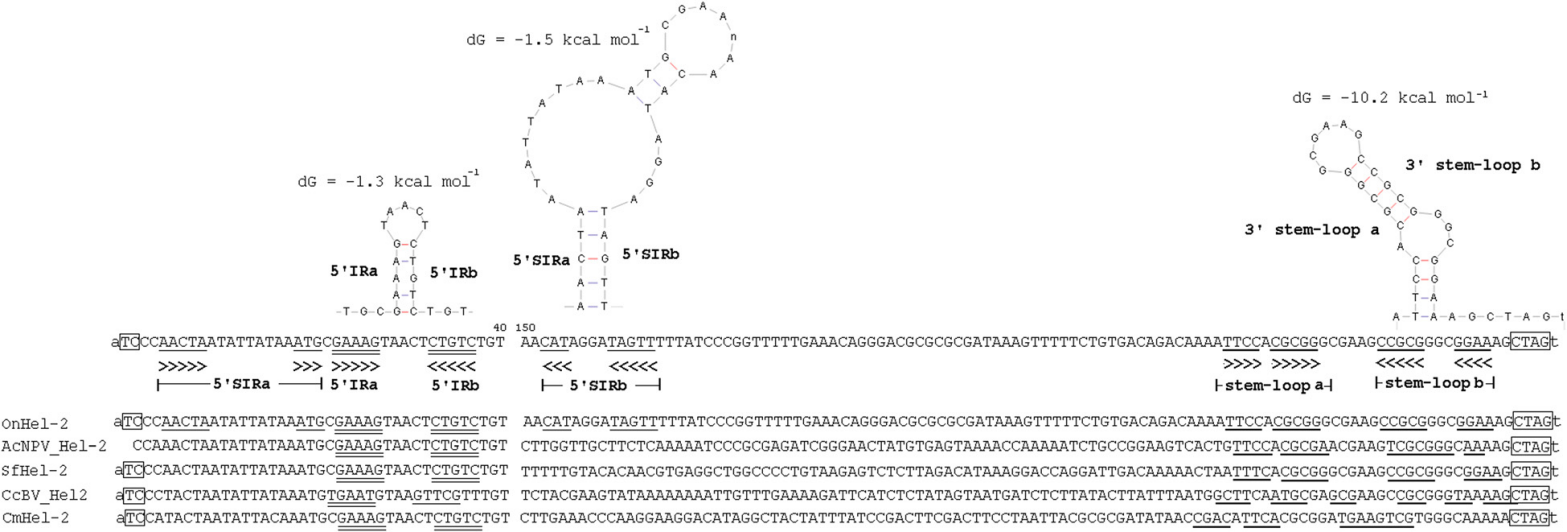
***Helitron*** **secondary structure elements.** Annotation of secondary structures predicted in the Hel-2 *Helitrons*, including formation of a 5′-inverted repeat (5′-IR), 5′- subterminal inverted repeat (5′-SIR), and a 3′-terminal stem-loop which are underscored with arrows indicating direction of inverted repeats. *Helitron* 5′-TC and 3′-CTAG termini are enclosed in boxes and flanking A/T dinucleotides are in small caps. dG = Gibbs Free energy.

Despite the prior prediction of homology among this *Helitron* family, the mode of propagation across the lepidopteran lineage has not been investigated. A broad homology-based search of contemporary DNA sequence resources identified 488, 735, 661, and 73 homologous *Helitrons* respectively in GenBank nr, dbEST, dbGSS, HTGS, and TSA database accessions (Additional file 2). DNA sequence identities ≥ 65.7% were observed to the OnHel-2 query among accession from species of insects from the Orders Lepidoptera (*n* = 24), Hymenoptera (2), Coleoptera (2), Hemiptera (1), and Phasmida (1), as well as sequences from insect bracovirus and baculovirus (5), and plant species (5). In many instances full-length elements could not be identified from GenBank accessions due to the short nature of many entries and is a limitation of many dbGSS or dbEST accessions. The inability to define full-length *Helitrons* also likely resulted when one or both termini remained cryptic, which was presumably due to mutations caused by the integration of other TEs or substitution mutation that obscured 5′-TC and 3′-CTRR motifs. Alternatively, sequence gain has been observed among *Helitrons* from maize [11,12] as well as Lepidoptera [5,13] such that potential acquisition of large sequences may have negatively affected the detection of *Helitron* termini, but this was not investigated further in this study. Regardless, *Helitron*-like 5′-TC and 3′-CTRR termini and flanking AT dinucleotides [1], and 3′-stem-loops (hairpins) were predicted upstream of the 3′-CTRR termini of this *Helitron* family (Figure 1). Additional secondary structure elements were predicted for representative OnHel-2 and BmHel-2 *Helitrons* including a 5′-inverted repeat (IR), subterminal inverted repeats (SIRs) that were originally described from drosopholid [4] and lepidopteran *Helitrons* [13,16]. Hel-2 copies from insects were distinctive in that a variable-length tetranucleotide repeat microsatellite (CTGT)_n_ was present downstream of the 5′-SIR. These results suggest that Hel-2-like *Helitrons* are a prevalent TE lineage in the genomes of Lepidoptera, and are analogous to other *Helitrons* from Lepidoptera in that they show broad distributions and share extensive sequence homology among copies integrated in the genomes of different species [5,13,16].

### Horizontal transfer of Hel-2 *Helitrons* among insects and viruses

TEs enter a genome lineage either through vertical inheritance from a progenitor species or by HTT. The “patchy distribution” of a TE among genomes of a taxa is typically used to support the historical occurrence for HTT. *Helitron* copy numbers were predicted to vary ~33-fold between the genomes of *H. melpomene* and *D. plexippus* [5], which suggests that TEs have had variable success at colonizing the genomes of Lepidoptera, or species with low observed *Helitron* densities have only recently experienced invasion by this TE. The variation in *Helitron* copy number between species is likely subject to stochastic processes of genetic drift when the TE initially invades a genome [35,36], whereby newly acquired TEs can be lost or retained at low copy if transposition is not robust or germline transformation does not occur. Selection may also be a major constraint on TE propagation in a genome, where an equilibrium develops between TE gain and loss [37,38]. Due to potential loss of a TE from previously invaded genomes, evidence consisting of a “patchy distribution” across species of a taxonomic group may, in general, not be reliable evidence in support of HTT. Furthermore, sparse sequence resources for many species of Lepidoptera result in unreliable and inappropriate estimates of *Helitron* presence or absence using a bioinformatic approach, but may improve as additional genome resources become available [39].

**Figure 2:**
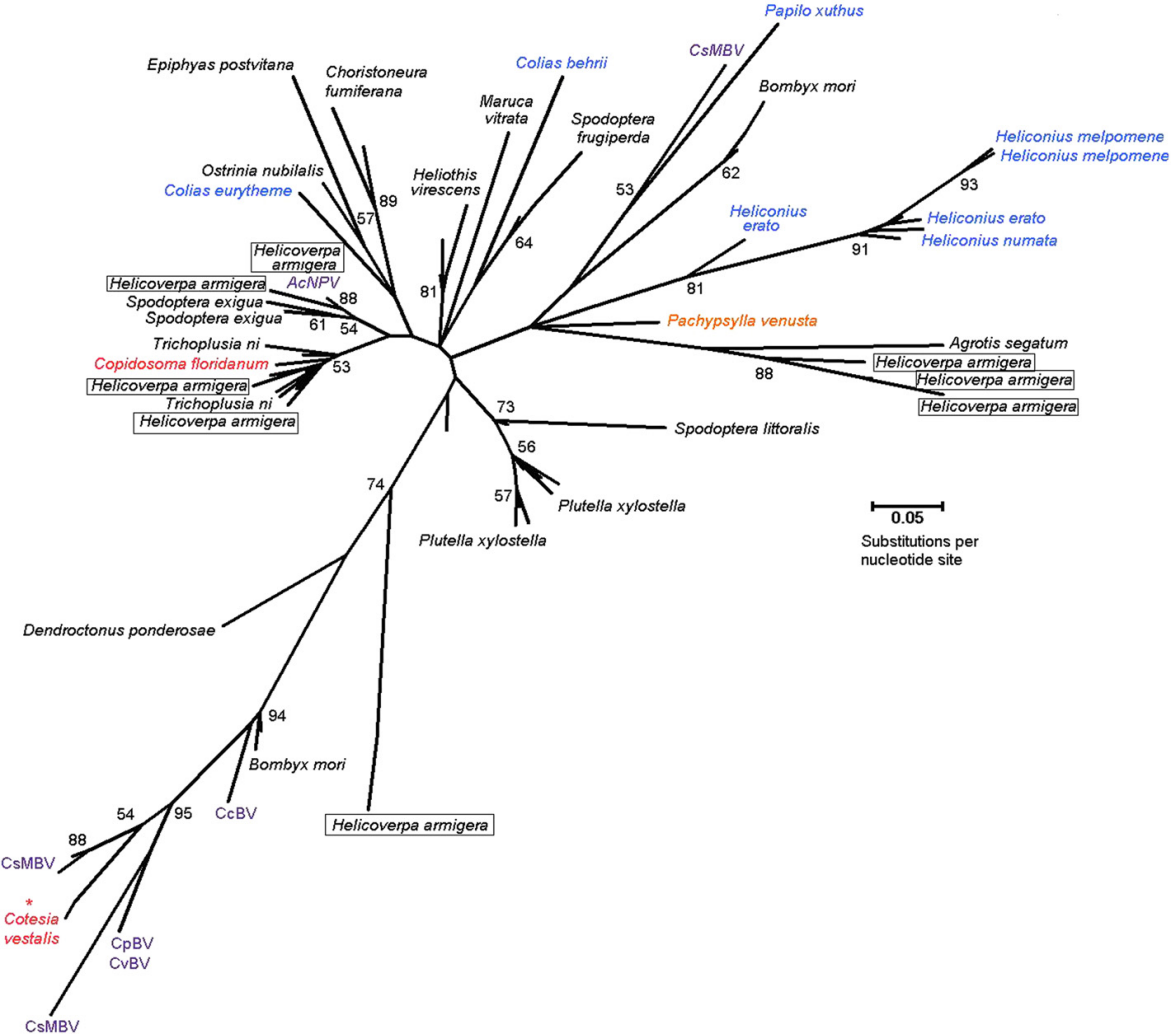
**Phylogenetic relationship among Helitron-like Hel-2 elements horizontally transferred among insects and viruses.** Maximum likelihood (ML) estimation among Hel-2 elements in genomes of moths, butterflies, hymenopteran parasitoids, a hemipteran gall former, and insect viruses. using the Tamura-3-Parameter model and Gamma shape parameter of 2.249. Bootstrap support is indicated at each node of the consensus tree when values were ≥ 50%, whereas unresolved branches exist for all other instances. Resulting tree has a Log Likelihood = − 1908.7; total branch length of 5.438. Species definitions: Moths: Family Bombycidae, Bombyx mori; Family Crambidae, Ostrinia nubilalis and Maruca vitrata; Family Noctuidae, Agrotis sgatum, Helicoverapa armigera, Heliothis virescens, Spodoptera frugiperda, S. exigua, and S. littoralis; Family Tortricidae, Choristoneura fumiferana and Epiphyas postvittana, Family Plutellidae, Plutella xylostella Butterflies: Family Pieridae, Colias eurytheme and C. behrri, Family Papilionidae, Papilio xuthus; Family Nymphalidae, Heliconius erato, H. numata and H. melpomene. *C. vestalis TSA submissions determined to share flanking sequence with CvBV segements and are suggested to comprise transcripts derived from viral integrations.

When vertical movement of DNA descends to progenitor genomes at the point of complete speciation, the subsequent bifurcating relationships between TE and species phylogenies tend to be highly correlated. In contrast, genome invasion by HTT violates these correlations due to the crossing of species boundaries, wherein rates of TE divergence no longer parallel genome divergence of the hosts [40]. HTT essentially causes introgression of TE sequences across affected species and results in incomplete lineage sorting that tends to be problematic for phylogenetic algorithms. Indeed, phylogenetic reconstruction of the Hel-2 *Helitron* resulted in few well supported nodes (bootstrap values ≥ 50%), but generally did reflect some of the broad relationships among species of Lepidoptera [33] in that Hel-2 copies in the genus *Heliconius* in the Superfamily Papilionidea (true butterflies) are well supported (Figure 2). The phylogenetic tree also showed distinct clades for Hel-2 copies from the species *Plutella xylostella*, *Heliothis virescens*, and *Choristoneura fumiferana*, which may be a consequence of more recent invasions of these genomes where Hel-2 copies are relatively homogenized due to insufficient time for substitution mutations to have accumulated. In contrast, two well supported clades with bootstrap values ≥ 52 containing Hel-2 copies from the genomes of multiple species from the Family Noctuidae (Figure 2) may indicate that Hel-2 copies are more closely related between noctuid species compared to within a species. This is highlighted by instances where Hel-2 *Helitrons* from the genome of *Helicoverpa armigera* show higher interspecific similarity and cluster with Hel-2 copies in other noctuid species including *T. ni*, and might provide partial evidence for HTT. Lower nucleotide divergence was predicted between these *H. armigera* and *T. ni* Hel-2 *Helitrons* (Additional file 3A) compared to RpS5 housekeeping gene sequences (Additional file 3B; Table 1). Albeit narrow in scope, this suggests a more recent ancestry among TEs compared to the genomic background of these insect species, and in combination with phylogenetic analysis provide putative evidence of HTT. The inability to completely resolve this phylogeny was not surprising due to the short DNA sequence data inherent to non-autonomous *Helitrons*, which compared to analogous reconstructions using derived amino acid, are more prone to the effects of forward and back mutation or convergence over longer evolutionary time frames. Additionally, HTT is expected to cause incomplete lineage sorting which causes known problems with phylogentic analyses [41]. Thus, the construction of phylogenies from TE sequence data may become increasingly confounded with increasing evolutionary time and increasing incidence of HTT.

**Table 1:** **Comparative divergence between** ***Helitron*** **and host genomic sequence**

**GenBank accession**	**Species**	**1.**	**2.**	**3.**	**4.**	**5.**	**6.**
JX683313.1	1. *O. nubilalis*	100.00	79.01	76.77	75.45	**83.11**	67.77
JN383812.1	2. *P. xylostella*	81.66	100.00	78.45	75.33	80.09	73.63
AY837869.1	3. *T. ni*	85.21	85.89	100.00	**88.62**	78.47	74.02
KF624964.1	4. *H. armigera*	85.89	83.60	**86.60**	100.00	77.46	75.90
FE273699.1	5. *C. fumiferana*	**82.27**	84.22	82.09	83.16	100.00	63.84
AY769319.1	6. *B. mori*	82.39	80.60	83.98	82.19	79.43	100.00

Matrix showing nucleotide identity between Helitrons (above diagonal) and genomic sequences (ribosomal protein S5; below diagonal). Instances of higher similarity between TEs compared to host genomes are bolded. Sequences used in comparisons are provided in Additional file 3.

**Figure 3:**
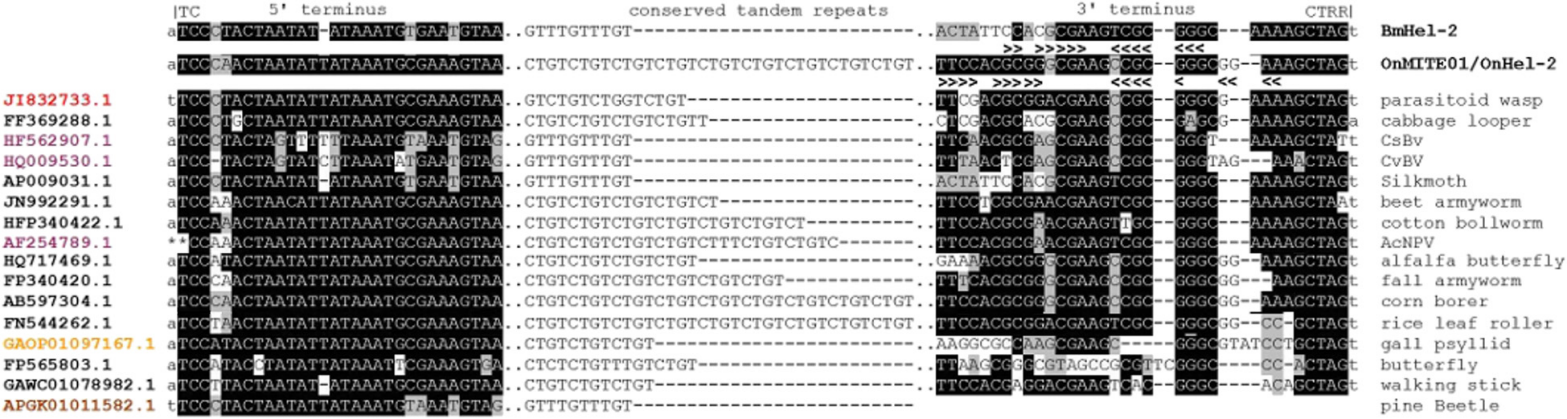
**Nucleotide conservation at 5′- and 3-′ termini among non-autonomous Hel-2 Helitrons from insect and insect virus genomes.** Alignment shows highly conserved nucleotides (highlighted black), and most prevalent alternate nucleotide (highlighted grey), and gaps are represented as a dash (−). Length and unit of internal tandem repeat arrays are shown. GenBank accession numbers and common names are respectively provided on the left and right of each sequence. Full-length annotated Hel-2 Helitrons used in this partial sequence alignment are provided in Additional file 4.

It is becoming generally accepted that TEs are transferred among eukaryotes with taxonomic close relationships, such as among genomes of insect [42], plant [43] and mammalian species [44], or among species that share pathogenic, parasitic or endosymbiont vectors [17,21]. Comparative genomic approaches have become feasible through the accumulation of increasing amounts of genome sequence data and provide additional, and arguably more reliable, evidence for detecting the occurrence and possible modes of HTT. Prior sequence homology evidence showed that a *mariner*-like element had recently been transferred between the genomes of an insect host, *Adoxophyes honmai* (Lepidoptera: Tortricidae), and it’s parasitoid wasp, *Ascogaster reticulatus* (Hymenoptera: Braconidae), which suggested the “intimate” relationship between these species facilitated HTT by an unknown mechanism [45]. Additionally comparative genomic evidence documented the transposition and integration of a reptilian short interspersed nuclear element (SINE) into the genome of a dsDNA taterapox virus (TATV), which suggested that viruses are capable of acquiring host TEs and could potentially be a vector for HTT [46]. Two pervasive *Helitrons* from species of Lepidoptera were reported to have integrated into the genomes of dsDNA polydnaviruses (PDVs), the *Cotesia plutella* bracovirus (CpBV) and the *C. sesamiae* bracovirus (CsMBV) [17]. Analogously, results from this study detected novel Hel-2 elements integrated in the genomes of CpBV, CsMBV as well as *C. congregata* BV (CcBV) and *C. vestalis* BV (CvBV; Figure 3; Additional file 4) which were novel and not described in prior studies [17]. Similar to copies in the genomes of lepidopteran insects, *Helitron*-like termini and (CTGT)_n_ or (GTTT)_n_ repeats were also annotated in most viral and parasitoid wasp derived elements (Additional file 5). This accumulating evidence suggests that the biology and mode of PDV infection may facilitate HTT among related Lepidoptera. Specifically, the genomes of PDVs are segmented and integrated into the genomes of their primary hosts (parasitoid wasps; Hymenopteran: Ichneumonidae and Braconidae), but these viral genome segments also exist as free circular dsDNAs that become enclosed into transmissible viral particles [47,48]. PDV virons are produced in specialized ovarian cells of the parasitoid wasps and are transmitted by female wasps during oviposition into larval Lepidoptera. Infecting PDVs act to suppress the immune response of lepidopteran larvae, and thus have evolved a mutual relationship with the wasps [49]. PDV DNA enters the nucleus of infected cells wherein viral gene products may down-regulate larval immune response genes and could potentially be targeted for integration by actively transposing host *Helitrons*. Our data analysis suggest that a Hel-2 element integrated into the genome of the parasitoid chalcid wasp, *Copidosoma floridanum*, also shows a close phylogenetic relationship with Hel-2 elements in the genome of its primary host, *T. ni* [50] (Figure 2). This is suggestive but not conclusive evidence that HTT may occur through tri-tropic associations between parasitoids wasps, PDVs and host Lepidoptera, where HTT of another *Helitron* between *T. ni* and *C. floridanum* lacked evidence of PDV involvement [27].

**Figure 4:**
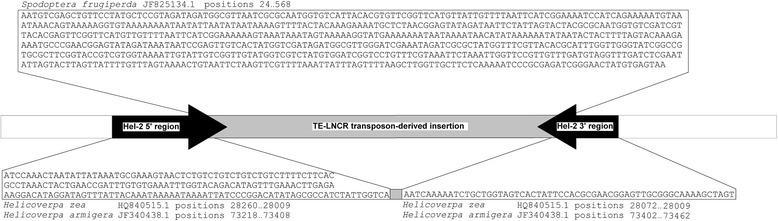
**Structure of the** ***Autographa californica*** **nucleopolyhedrovirus (AcNPV) element.** The element inserted into the AcNPV genome following serial passage through cell lines from the noctuid insects *Spodoptera frugipera* and *Trichoplusia ni*. The mobile element contains termini that show ≥ 95.4% nucleotide similarity to Hel-2 *Helitrons* integrated into the genomes of *Helicoverpa* sp. (Lepidoptera: Noctuidae) and are represented ass black arrows. The central region of the element is putatively derived from nested insertion of a TE-LNCR transposon (grey bar) initially described from *S. frugiperda* (Lepidoptera: Noctuidae; see Discussion for appropriate citations).

In addition to the detection of Hel-2 integrations in PDVs, databases search results from the current study show that a mutant form of *Autographa californica* nucleopolyhedrovirus (AcNPV) contained a Hel-2 *Helitron* integration (Additional file 2). AcNPV particles directly infect larval Lepidoptera including the Alfalfa Looper, *Autographa californica* (Lepidoptera: Noctuidae) and other species in the lepidopteran family Noctuidae. This previously described integration encompassed a 1006 bp regions of the AcNPV mutant vsk-1d1 that was reported to have spontaneously inserted into the *Pst*I-1 region between ORFs 28 and 29 of the AcNPV genome after serial passage through *T. ni* and *S. frugiperda* cell lines [28,51]. Our analysis showed that the 1006-bp AcNPV genome insertion showed 5′-TCC and 3′-CTAG terminal *Helitron* motifs, a predicted 3′-stem-loop as well as ≥ 83.2% sequence identity with Hel-2 *Helitrons* from *T. ni* and *S. frugiperda* (Figure 1). More detailed analysis of the 1006-bp Hel-2 from AcNPV suggests that the mobile element may be comprised of 5′- and 3′- Hel-2 termini interrupted by an approximately 577-bp region with homology to a *S. frugiperda* transposon named TE-LNCR [52] which accounts for increased size compared to other Hel-2 elements. Moreover, the termini of the AcNPV Hel-2 element show 95.63 and 95.37% nucleotide identity to *Helicoverpa zea* and *H. armigera* Hel-2 elements (Figure 4). Given that these two insect integrated elements shared 97.58% nucleotide identity, might indicate that this viral insertion element was likely derived from an insect genome. Even though portions were annotated using genome data from different insect species, this likely does not necessarily suggest that the AcNPV element has acquired this chimeric structure as a result of HTT but could also be result of the current inadequacy of genome sequence data. Although prior instances have documented the transposition of lepidopteran TEs into viruses [17,53,54], integration of Hel-2 into AcNPV after serial passage through permissive hosts provides a rare instance where HTT was observed in real time. In addition to this direct evidence of Hel-2 integration into AcNPV, the phylogenetic relationships of PDV and AcNPV *Helitrons* are intertwined with Hel-2 elements from insects. Specifically, strong bootstrap support was obtained for nodes defining close relationships between Hel-2 *Helitrons* from AcNPV and *Helicoverpa armigera* and *Spodoptera exigua* (Figure 2), and the Hel-2 element in AcNPV is comparatively more distant from those integrated into PDVs. Similarly, *Helitrons* from PDVs, and the lepidopteran species *B. mori*, *Cotesia vestalis*, and *Papilo xuthus* formed a well-supported cluster. It should be noted that Hel-2 copies identified in TSA accessions from the wasp *C. vestalis* shared strong sequence identity upstream of the 5′-TC and downstream of the 3′-CTAG termini with the PDV, CvBV, which was interpreted to indicate that the sequence were actually derived from an infecting virus (data not shown). In contrast, sequence flanking the Hel-2 copy identified from a TSA entry from the parasitoid wasp *Copidosoma floridanum* (Hymenoptera: Encyrtidae) show no homology to known virus DNAs, although origin from yet undescribed viruses cannot be ruled out. In conjunction with prior research outlined above, the comparative genomic data in this study indicate that viral genomes likely have acquired mobile elements from their insect hosts, with the assumption that cross-infection of related species by the virus and subsequent re-mobilization of the TE can result in HTT.

## Conclusions

Only recently has HTT been shown to occur between more distantly related taxa, where unknown mechanisms resulting from close associations between organisms have been implicated. For example, TEs from the DNA transposon hAT superfamily were shown to retain high levels of sequence homology among Chordates, Arthropods, Mollusks, and Platyhelminths, and provided estimates of genome invasion times that predated speciation as evidence for HTT [26]. Transfer of hAT transposons appeared to occur when host-parasite interactions present opportunities for exchange of cellular and genetic material, such as between the blood feeding insect *Rodnius prolixus* and mammals, or the pond snail *Lymnaea stagnalis* and vertebrates [26]. Both of these interactions involve an intermediate vector; the trypanosome *Trypanosoma cruzi* vectored by *R. prolixus*, and parasidic flatworms (trematodes) that are transmitted by *L. stagnalis*. This vector-dependent mechanism of HTT may be considered analogous to the viral-mediated horizontal transfer of *Helitrons* among Lepidoptera detected here and previously [17]. These data also suggest that HTT can occur across species of distant evolutionary relationships, and that the resulting spread of mobile elements into naive species may dramatically reshape genome structure and function [30]. Many further investigations as well as evidence of HTT of other TEs are definitely warranted before mechanisms or vectoring agents can be proposed.

Data presented here suggests that genetic information can be exchanged among highly diverged species. Although viruses were previously shown to vector *Helitrons* among insects and parasites have been implicated in the movement of TEs among tetrapods, evidence presented here may be the initial annotation of real-time HTT between insect and insect viral genomes. Although the mechanisms remain unclear and shear number of insect-derived TE integrations predicted in parasite genomes appears infrequent, the consequences of this movement may be substantial given the ancestral relationships between insects and their associated viruses. This suggests that HTT mediated by insect-pathogen interactions might affect the genome evolution of the species involved through mobile DNA transfer.

## Methods

### Prediction and annotation of highly conserved *Helitron* integrations

Previously identified TEs, the BmHel-2 from the genome of the silkmoth, *Bombyx mori* [5] and a 239 bp miniature inverted repeat transposable element (MITE01) characterized in *O. nubilalis* BAC inserts [32] (nucleotide positions 29 to 273 of GenBank accession ET217030.1) comprise highly similar DNA elements. The DNA sequence similarity among MITE01 and BmHel-2 sequences were estimated by alignment between *O. nubilalis* from GenBank accessions AB597304.1 (positions 1429 to 1179) and EF396411.1 (685 to 943), and *B. mori* accessions AP009003.1 (89798 to 90133) and AP009031.1 (54931 to 54604) using the ClustalW algorithm (Gap penalty = 10; Gap Extension - 0.2). BmHel-2 contains sequence features that led to classification as a *Helitron*-like TE [5], but OnMITE01 was previously described as a putative miniature inverted repeat transposable element (MITE) [9]. Additional annotation of MITE01 included prediction of putative primary sequence and secondary structure features consistent with other *Helitron*-like TEs from Lepidoptera using methods described previously [13,16] using the Mfold DNA server [55] (http://mfold.rna.albany.edu/?q=mfold/dna-folding-form). Novelty of MITE01 and BmHel-2 TEs was investigated by using respective sequences as queries against all sequence sources in RepBase (http://www.girinst.org/censor/index.php) [56].

A sequence homology approach was used to predict *Helitron* integrations in publically available genome sequence resources, and relied upon sequence conservation among non-autonomous *Helitron*-like TEs among Lepidoptera [13,16,17,32]. In this instance, the MITE01 and BmHel-2 *Helitron* sequences were used as queries to search the non-redundant (nr) nucleotide database (nr), expressed sequence tag (dbEST), genome survey sequence (dbGSS), high throughput genome sequence (HTGS), and transcriptome sequence assembly (TSA) databases housed at the National Center for Biotechnology Information (NCBI). Query of whole genome sequences from *B. mori*, *D. plexipus*, *H. melponene*, and *M. sexta* were previously conducted for BmHel-2 [5], and were not repeated in this study. All web-based queries used the blastn algorithm parameters adjusted for divergent sequences (discontinuous blastn) [57], and database search results were filtered as described by Coates et al. [13]. Results were further filtered for those with homology (65% sequence identity and *E*-values ≤ 10^−5^) to the entire query length, or modified to ≥ 50 bp to either the 5′- or 3′- ends to capture partial sequence data or copies that have undergone insertion mutations caused by other TEs.

### Horizontal transfer of Hel-2 *Helitrons* among insects and viruses

Incongruence between established interspecies relationships (“species trees”) and TE phylogenies can provide evidence for the HT of elements among genomes. A consensus alignment among 77 Hel-2 elements indentified from searches of GenBank nr, dbEST, dbGSS, HTGS and TSA databases was constructed using the ClustalW algorithm in the MEGA 5.2.1 alignment Suite [58] using default parameters, except the gap penalty was set to 10.0. Manual adjustments were made to the multiple sequence alignment, and converted to .meg format. The MEGA 5.2.1 software package [58] was further used to determine the optimal model of sequence evolution and gamma shape parameter. A Maximum Likelihood (ML) tree was constructed using the Tamura-3-parameter model with completed deletion of gapped data, and a consensus tree was reported from among random trees constructed from 1000 bootstrapped pseudoreplicates of the aligned sequence data [59] using MEGA 5.2.1 software package [58] (branch support ≥ 50% reported). Incomplete lineage sorting, convergence or other phenomenon may likely influence the resulting largely unresolved Hel-2 phylogeny, such that evolutionary relationships among HTT events could not be ascertained. Species relationships among Families of Lepidoptera were obtained from published estimates [33], and used to make *ad hoc* comparisons to the obtained Hel-2 phylogeny for qualitative description of distributions of lepidopteran species in well-supported TE clades.

## Additional files

Additional file 1: Figure S1. Estimation of sequence similarity between Hel-2 *Helitrons* from *Ostrinia nubilalis* (previously named OnMITE01; Coates et al. [31]) and *Bombyx mori* (Han et al. [5]) identified from GenBank accessions. A) Alignment of Hel-2 *Helitron* sequences from *Bombyx mori* and *Ostrinia nubilalis* using the CLUSTAL 2.1 algorithm. Flanking a/t dinucleotides from host genomic DNA are in small caps, *Helitron*-like 5′-TC and 3′-CTAG termini are underlined, and bases involved in formation of the 3′-stem-loop are indicated with arrows. Homologous nucleotides positions are highlighted and gaps are indicated as dashes (−). B) Percent identity matrix between *B. mori* and *O. nubilalis* Hel-2 *Helitrons*. Additional file 2: Table S1. Table of BLASTn search results and associated GenBank nr database accessions with sequence similarity to Hel-2 *Helitron* queries. Additional file 3: Data S1. Fasta formatted data used for estimation of sequence divergence between genomic housekeeping genes and Hel-2 elements among Lepidoptera. Additional file 4: File S3. GenBank accessions used in Figure 1 of the main text annotated with Hel-2 sequence features of a 5′-TC and 3′-CTAG, and (CTGT)_n_ or (GTTT)_n_ microsatellite repeats are indicated. Flanking genomic DNA is in small caps. Additional file 5: Figure S2. Full GenBank sequence accessions containing Hel-2 *Helitrons* integrations in the genomes of A) lepidopteran infecting viruses, and B) parasitoid wasps. *Helitron*-like 5′-TC and 3′-CTAG, and (CTGT)_n_ or (GTTT)_n_ microsatellite repeats are indicated. Flanking genomic DNA is in small caps.

## Abbreviations

HTT: Horizontal transposon transfer
DNA: Deoxyribonucleic acid
TE: Transposable element
MITE: Miniature inverted repeat transposable element
RCR: Rolling circle replication
ssDNA: Single stranded deoxyribonucleic acid
RPA: Replication protein A
mya: Million years ago
nr: Non-redundant
dbEST: Database of expressed sequence tags
dbGSS: Database of genome survey sequences
HTGS: High throughput genome sequence
TSA: Transcriptome sequence assembly
SIR: Subterminal inverted repeat
RpS: Ribosomal protein small subunit
SINE: Short interspersed nuclear element
CpBV: *Cotesia plutella* bracovirus
CsMBV: *Cotesia sesamiae* bracovirus
CcBV: *Cotesia congregate* bracovirus
CsBV: *Cotesia vestalis* bracovirus
PDV: PolyDNA virus
dsDNA: Double-stranded DNA
AcNPV: *Autographa californica* nucleopolyhedrovirus

## References

[1] Kapaitonov VV, Jurka J (2001). Rolling-circle transposons in eukaryotes. Proc Natl Acad Sci U S A.

[2] Lal SK, Giroux MJ, Brendel B, Vallejos CE, Hannah LC (2003). The maize genome contains a *Helitron* insertion. Plant Cell.

[3] Pritham EJ, Feschotte C (2007). Massive amplification of rolling-circle transposons in the lineage of the bat *Myotis lucifugus*. Proc Natl Acad Sci U S A.

[4] Yang HP, Barbash DA (2008). Abundant and species-specific DINE-1 transposable elements in 12 *Drosophila* genomes. Genome Biol.

[5] Han MJ, Sen YH, Xu MS, Liang HY, Zhang HH, Zang Z (2013). Identification and evolution of the silkworm *Helitrons* and their contribution to transcripts. DNA Res.

[6] Liu H, Fu Y, Li B, Yu Z, Zie J, Cheng J (2011). Widespread horizontal gene transfer from circular single-stranded DNA viruses to Eurkaryotic genomes. BMC Evol Biol.

[7] Gallivotti A, Zhao Q, Kyozuka J, Meeley RB, Ritter MK, Doebley JF (2004). The role of barren stalk1 in the architecture of maize. Nature.

[8] Choi JD1, Hoshino A, Park KI, Park IS (2007). Spontaneous mutations caused by a *Helitron* transposon, Hel-It1, in morning glory, *Ipomoea tricolor*. Plant J.

[9] Inagaki S, Nakamura K, Morikami A (2009). A link among DNA replication, recombination, and gene expression revealed by genetic and genomic analysis of *TEBICHI* gene of *Arabidopsis thaliana*. PLoS Genet.

[10] Morgante M, Brunner S, Pea G, Fengler K, Zuccolo A, Rafalski A (2005). Gene duplication and exon shuffling by *helitron*-like transposons generate intraspecies diversity in maize. Nature Genet.

[11] Barbaglia AM, Klusman KM, Higgins J, Shaw JR, Hannah LC, Lal SK (2012). Gene capture by *Helitron* transposons reshuffles the transcriptome of maize. Genetics.

[12] Lai J, Li Y, Messing J, Dooner HK (2005). Gene movement by *Helitron* transposons contributes to haplotype variability of maize. Proc Natl Acad Sci U S A.

[13] Coates BS, Hellmich RL, Grant DM, Abel CA (2012). Mobilizing the genome of Lepidoptera through novel sequence gains and end creation by non-autonomous *Lep*1 *Helitrons*. DNA Res.

[14] Galagan JE, Calvo SE, Cuomo C, Ma LJ, Wortman JR, Batzoglou S (2005). Sequencing of *Aspergillus nidulans* and comparative analysis with *A. fumigatus* and *A. oryzæ*. Nature.

[15] Ray DA, Feschotte C, Pagan HJT, Smith JD, Pritham EJ, Arensburger P (2008). Multiple waves of recent DNA transposon activity in the bat, *Myotis lucifugus*. Genome Res.

[16] Coates BS, Sumerford DV, Hellmich RL, Lewis LC (2010). A *Helitron*-like transposon superfamily from Lepidoptera disrupts (GAAA)_n_ microsatellites and is responsible for flanking sequence similarity within a microsatellite family. J Mol Evol.

[17] Thomas J, Schaack S, Pritham EJ (2010). Pervasive horizontal transfer of rolling-circle transposons among animals. Genome Biol Evol.

[18] Thomas J, Vadnagara K, Pritham EJ (2014). DINE-1, the highest copy number repeats in *Drosophila melanogaster* are non-autonomous endonuclease-encoding rolling-circle transposable elements (*Helentrons*). Mob DNA.

[19] Silva JC, Kidwell MG (2000). Horizontal transfer and selection in the evolution of P elements. Mol Biol Evol.

[20] Coates BS, Hellmich RL, Lewis LC (2002). Nuclear small subunit rRNA group I intron variation among *Beauveria* spp provide tools for strain identification and evidence of horizontal transfer. Curr Genet.

[21] Vavre F, Fleury F, Lepetit D, Fouillet P, Bouletreau M (1999). Phylogenetic evidence for horizontal transmission of *Wolbachia* in host-parasitoid associations. Mol Biol Evol.

[22] Klasson L, Kambris Z, Cook PE, Walker T, Sinkins SP (2009). Horizontal gene transfer between *Wolbachia* and the mosquito *Aedes aegypti*. BMC Genomics.

[23] Waterhouse DF, Norris KR (1987). Biological control – pacific prospects.

[24] Polaszek A, Walker AK (1991). The *Cotesia flavipes* species complex: parasitoids of cereal stem borers in the tropics. Redia.

[25] Houch MA, Clark JB, Peterson KR, Kidwell MG (1991). Possible horizontal transfer of *Drosophila* genes by the mite *Proctolaelaps regalis*. Science.

[26] Zhang HH, Feschotte C, Han MJ, Zhang Z (2014). Recurrent horizontal transfers of Chapaev transposons in diverse invertebrate and vertebrate animals. Genome Biol Evol.

[27] Guo X, Gao J, Li F, Wang J (2014). Evidence of horizontal transfer of non-autonomous Lep1 *Helitrons* facilitated by host-parasite interactions. Scientific reports.

[28] Kumar S, Miller LK (1987). Effects of serial passage of *Autographa californica* nuclear polyhedrosis virus in cell culture. Virus Res.

[29] Gilbert C, Schaack S, Pace JK, Brindley PJ, Feschotte C (2010). A role of host-parasite interactions in the horizontal transfer of transpososons across phyla. Nature.

[30] Brookfield JF, Badge RM (1997). Population genetics models of transposable elements. Genetica.

[31] Yang HP, Tl H, You TL, Yang TH (2006). Genome-wide comparative analysis of the highly abundant transposable element DINE-1 suggests a recent transpositional burst in *Drosophila yakuba*. Genetics.

[32] Schaack S, Gilbert C, Feschotte C (2010). Promiscuous DNA: horizontal transfer of transposable elements and why it matters for eukaryotic evolution. Trends Ecol Evol.

[33] Coates BS, Sumerford DV, Hellmich RL, Lewis LC (2009). Repetitive genomic elements in a European corn borer, *Ostrinia nubilalis*, bacterial artificial chromosome library were indicated by bacterial artificial chromosome end sequencing and development of sequence tag site markers: implications for lepidopteran genomic research. Genome.

[34] Wahlberg N, Wheat CW, Peña C (2013). Timing and Patterns in the taxonomic diversification of Lepidoptera (Butterflies and Moths). PLoS One.

[35] Lavoie CA, Platt RN, Novick PA, Counterman BA, Ray DA (2013). Transposable element evolution in *Heliconius* suggests genome diversity within Lepidoptera. Mob DNA.

[36] Brookfield JFY (1991). Models of repression and transposition in PM hybrid dysgenesis by P cytotype and by zygotically encoded repressor proteins. Genetics.

[37] Charlesworth B, Lapid A, Canada D (1992). The distribution of transposable elements within and between chromosomes in a population of *Drosophila melanogaster*. II. Inferences on the nature of selection against elements. Genet Res.

[38] Charlesworth B, Sniegowski P, Stephan W (1994). The evolutionary dynamics of repetitive DNA in eukaryotes. Nature.

[39] i5K Consortium. The i5K Initiative (2013). Advancing arthropod genomics for knowledge, human health, agriculture, and the environment. J Heredity.

[40] Daniels SB, Peterson KR, Strausbaugh LD, Kidwell MG, Chovnick A (1990). Evidence for horizontal transmission of the P transposable element between *Drosophila* species. Genetics.

[41] Maddison WP, Knowles LL (2005). Inferring phylogeny despite incomplete lineage sorting. Syst Biol.

[42] Robertson HM, Lampe DJ (1995). Recent horizontal transfer of a mariner transposable element among and between Diptera and Neuroptera. Mol Biol Evol.

[43] Diao X, Freeling M, Lisch D (2005). Horizontal transfer of a plant transposon. PLoS Biol.

[44] Pace JK, Gilbert C, Clark MS, Feschotte C (2008). Repeated horizontal tranfer of a DNA transposon in mammals and other tetrapods. Proc Natl Acad Sci U S A.

[45] Yoshiyama M, Tu Z, Kainoh Y, Honda H, Shono T, Kimura K (2001). Possible horizontal transfer of a transposable element from host to parasitoid. Mol Biol Evol.

[46] Piskurek O, Okada N (2007). Poxviruses as possible vectors for horizontal transfer of retroposons from reptiles to mammals. Proc Natl Acad Sci U S A.

[47] Stoltz DB, Krell PJ, Summers MD, Vinson SB (1984). Polydnaviridae – a proposed family of insect viruses with segmented, double-stranded, circular DNA genomes. Intervirology.

[48] Espagne E (2004). Genome sequence of a polydnavirus: insights into symbiotic virus evolution. Science.

[49] Strand MR, Beckage NE, Drezen JM (2012). Polydnavirus gene products that interact with the host immune system. Parasitoid viruses: symbionts and pathogens.

[50] Strand MR (1989). Development of the polyembryonic parasitoid *Copidosoma floridanum* in *Trichoplusia ni*. Entomol Exp et Appl.

[51] McLachlin JR, Escobar JC, Harrelson JA, Clem RJ, Miller LK (2001). Deletions in the Ac-iap1 gene of the baculovirus AcMNPV occur spontaneously during serial passage and confer a cell line-specific replication advantage. Virus Res.

[52] Stanojcic S, Gimenez S, Permal E, Cousserans F, Quesneville H, Fournier P (2011). Correlation of LNCR rasiRNAs expression with heterochromatin formation during development of the holocentric insect *Spodoptera frugiperda*. PLoS One.

[53] Jehle JA, Nickel A, Vlak JM, Backhaus H (1998). Horizontal escape of the novel Tc1-like lepidopteran transposon TCp3.2 into *Cydia pomonella* granulovirus. J Mol Evol.

[54] Friesen PD, Nissen MS (1990). Gene organization and transcription of ted, a lepidopteran retrotransposon integrated within the baculovirus genome. Mol Cell Biol.

[55] Zuker M (2003). Mfold web server for nucleic acid folding and hybridization prediction. Nucl Acids Res.

[56] Kohany O, Gentles AJ, Hankus L, Jurka J (2006). Annotation, submission and screening of repetitive elements in Repbase: RepbaseSubmitter and Censor. BMC Bioinformatics.

[57] Altschul SF, Gish W, Miller W, Myers EW, Lipman DJ (1990). Basic local alignment search tool. J Mol Biol.

[58] Tamura K, Peterson D, Peterson N, Stecher G, Nei M, Kumar S (2011). Molecular evolutionary genetics analysis using maximum likelihood, evolutionary distance, and maximum parsimony methods. Mol Biol Evol.

[59] Felsenstein J (1985). Confidence limits on phylogenies: an approach using the bootstrap. Evolution.

