# Supplementary material for: Horizontal transfer of a non-autonomous Helitron among insect and viral genomes
Source: BMC Genomics. 2015 Feb 27;16(1):137. doi: 10.1186/s12864-015-1318-6 (PMC4344730; doi:10.1186/s12864-015-1318-6)
Supplement: Additional file 1: Figure S1. — Estimation of sequence similarity between Hel-2 Helitrons from Ostrinia nubilalis (previously named OnMITE01; Coates et al. [31]) and Bombyx mori (Han et al. [5]) identified from GenBank accessions. A) Alignment of Hel-2 Helitron sequences from Bombyx mori and Ostrinia nubilalis using the CLUSTAL 2.1 algorithm. Flanking a/t dinucleotides from host genomic DNA are in small caps, Helitron-like 5′-TC and 3′-CTAG termini are underlined, and bases involved in formation of the 3′-stem-loop are indicated with arrows. Homologous nucleotides positions are highlighted and gaps are indicated as dashes (−). B) Percent identity matrix between B. mori and O. nubilalis Hel-2 Helitrons. [file 12864_2015_1318_MOESM1_ESM.pdf]

Additional file 1: Figure S1. Estimation of sequence similarity between Hel-2 *Helitrons* from *Ostrinia nubilalis* (previously named OnMITE01; Coates et al. 2009) and *Bombyx mori* (Han et al. 2013) identified from GenBank accessions.

A) Alignment of Hel-2 *Helitron* sequences from *Bombyx mori* and *Ostrinia nubilalis* using the CLUSTAL 2.1 algorithm. Flanking a/t dinucleotides from host genomic DNA are in small caps, *Helitron*-like 5'-TC and 3'-CTAG termini are underlined, and bases involved in formation of the 3'-stem-loop are indicated with arrows. Homologous nucleotides positions are highlighted and gaps are indicated as dashes (-).

```
1) AP009003.1    aTCCCTACTAATAT-ATAAATGTGAATGTAAGTTTGTTTGTAACGCTTTCACGCGAAAAC 59
2) AP009031.1    aTCCCTACTAATAT-ATAAATGTGAATGTAAGTTTGTTTGTTACGCTTTCACGCGAAAAC 59
3) AB597304.1    aTCCCAACTAATATTATAAATGCGAAAGTAACTCTGTCTGT----CTGTC-TGT-----C 50
4) EF396411.1    aTCCCAACTAATATTATAAATGCGAAAGTAACTCTGTCTGT----CTGTC-TGT-----C 50

1) AP009003.1    TACTCGACCGATCATCATGAAACTTTGTACACATATTCTTGGAGGTATTAGAAGTAGCAT 119
2) AP009031.1    TACTCAACCGACCATCATGAAACTTTGTACACATATTGTTGGAGGTATTAGAAGTAACAT 119
3) AB597304.1    TGTCTGTCTGTCTGTC-TGT---------TACGCTTTCCCGC-----TTAAACCTCGCAA 95
4) EF396411.1    TGTCTGTCTGTCTGTC-TGTCTGTCTGT-TACGCTTTCCCGC-----TTAAACCTCGCAA 103

1) AP009003.1    AGGATACTTTTTATTAAGAAGAAAAAAAAT-TTTTTTACAAAAAATTAAAAAGTTGTTTG 178
2) AP009031.1    AGGATACTTTTTAGTATTAAAAAAATATATATTTTGTACGAAAAATAAAAAAATTGTTTG 179
3) AB597304.1    CCGAT----TTTGAT-----GAAA-------TTTGGCAT---------AGAGATAGTTTG 130
4) EF396411.1    CCGAT----TTTGAT-----GAAA-------TTTGGCAT---------AGAGATAGTTTG 138

1) AP009003.1    TCAAAAAATCTCAAAAATCTAGCTTCTTCAACGCCATCTACCGGTTCAGCAATGAAGTTC 238
2) AP009031.1    TCAAAAAATCTCAAAA-TCTAGCTTCTTCAACGCCATCTACCGGTTCAGTAATGAAGTTC 238
3) AB597304.1    ------AGTCCCGGGA-------------AAGAACAT----------AG-GAT--AGTTT 158
4) EF396411.1    ------AGTCCCGGGA-------------AAGAACAT----------AG-GAT--AGTTT 166

1) AP009003.1    CAATCCTGAGTACTGTAAATTAAGTGGGGGGGGGTAAAATGTAGCGTTATGCCAAAGTA 298
2) AP009031.1    CAATCCTGAGTACTGTAAATGAAGTCGCGGG----TAAAATTTAGCGTTATGCCAAAGTA 294
3) AB597304.1    TTATCCCGGTTTTTG-AAACAGGGACGCGCGCGA-TAAAGTTTTTC--TGTGACAGACAA 214
4) EF396411.1    TTATCCCGGTTTTTG-AAACAGGGACGCGCGCGA-TAAAGTTTTTC--TGTGACAGACAA 222

1) AP009003.1    ACTATTCCACGCGGACGAAGTCGCGGGCA-AAAGCTAGt 336
2) AP009031.1    ACTATTCCACGCG----AAGTCGCGGGCA-AAAGCTAGt 328
3) AB597304.1    A--ATTCCACGCGGGCGAAGCCGCGGGCGGAAAGCTAGt 251
4) EF396411.1    A--ATTCCACGCGGGCGAAGCCGCGGGCGGAAAGCTAGt 259
                       >>>>>>>>      <<<<<<<
```

B) Percent identity matrix between *B. mori* and *O. nubilalis* Hel-2 *Helitrons*.

GenBank accession	AP009003.1	AP009031.1	AB597304.1	EF396411.1
AP009003.1	100	93.88	66.67	66.15
AP009031.1	93.88	100	67.77	67.2
AB597304.1	66.67	67.77	100	100
EF396411.1	66.15	67.2	100	100
