# Supplementary material for: Horizontal transfer of a non-autonomous Helitron among insect and viral genomes
Source: BMC Genomics. 2015 Feb 27;16(1):137. doi: 10.1186/s12864-015-1318-6 (PMC4344730; doi:10.1186/s12864-015-1318-6)
Supplement: Additional file 3: Data S1. — Fasta formatted data used for estimation of sequence divergence between genomic housekeeping genes and Hel-2 elements among Lepidoptera. [file 12864_2015_1318_MOESM3_ESM.pdf]

Additional file 3: Data S1

A) Ribosomal protein S5 (RpS5) gene coding sequences used for the estimation of genomic sequence divergence.

>DQ988989.1_Onubilalis
ATGGCGGTGAACAACATTTCAAAAAAGCGAAAATTCGTCGGAGACGGAGTTTTCAAGGCGGAACTGAATG
AGTTCCTCACTCGGGAGCTGGCCGAAGATGGTTACTCCGGCGTGGAAGTGCGCGTCACCCCAACGCGGTC
GGAGATCATCATTATGGCCACCAGAACCCAGAGCGTACTGGGAGAGAAGGGTCGCAGAATTCGTGAACTA
ACCTCCGTGGTCCAGAAGCGTTTCAACATCCCTGAACAGTCTGTTGAGCTGTACGCTGAGAAAGTCGCCA
CTCGCGGTCTCTGCGCCATCGCGCAAGCCGAGTCTTTGAGATACAAACTTATCGGAGGTCTCGCTGTCCG
TCGTGCCTGCTATGGTGTGCTGAGGTTCATCATGGAGTCTGGCGCCCGCGGCTGCGAAGTGGTAGTGTCT
GGCAAGCTCCGTGGCCAGCGTGCCAAGTCGATGAAGTTCGTCGACGGCCTCATGATCCACTCCGGAGACC
CATGCAATGACTACGTCAACACCGCCACCAGGCACGTACTGCTCCGGCAAGGCGTGCTTGGAATCAAGGT
AAAGATCATGCTTCCGTGGGACCAGCAAGGCAAGAACGGCCCCAAGAAACCGCAGCCCGACCACATCCTG
GTCACGGAGCCCAAGGACGAGCCGGCGCCTCTCGAGCCTACCAACGAGCGTGCCATGGCGCCGCCGCCTG
CGCAGCCCGTAGCCGCCTCGGCATAA

>JN383812.1_Pxylostella
GGGGTCCTTTTACTCGTGTTCTGTTGACAGCGAGAGGTTATAATTGCTATTTCATAGCATAAAAGCTATA
ACAATGGCTGAAGAAAATTGGAATGACGAAGCCGTGGAGGCTGGCAGCATGGCTGTCGACAACATGCCCC
TGCCCCAGGCCGCTGACATCCCCGAAATCAAGCTGTTCGGCAGATGGAGTTGTTATGATGTCCAGGTTTC
GGACATGTCCCTGCAGGACTACATCTCCGTGAAGGAGAAGTACGCTAAATACCTCCCCCACTCCGCCGGC
AGGTACGCACACAAGCGCTTCCGTAAAGCGCAGTGCCCCATCGTCGAGCGTCTCACCAACTCCCTGATGA
TGCACGGACGCAACAACGGAAAGAAGTTGATGGCTGTTCGTATTGTGAAGCACGCTTTTGAGATCATCCA
TCTCCTAACTGGAGAGAACCCTCTCCAAGTGTTGGTCACAGCCATCATCAACTCTGGTCCCCGTGAAGAC
TCGACCAGAATCGGTCGCGCCGGTACCGTGCGTCGTCAGGCCGTGGACGTGTCTCCGCTGCGCCGAGTGA
ACCAGGCCATCTGGCTGCTGTGCACCGGAGCCCGTGAGGCTGCCTTCAGGAACATTAAGACCATTGCCGA
GTGTGTTGCTGACGAACTTATCAACGCCGCCAAGGGCTCGTCAAACTCATACGCCATCAAGAAGAAGGAT
GAACTCGAGCGTGTTGCCAAGTCTAACCGTTAATTTATATTAATTTGGGCAGGGCTCAATAAACTAAGAA
AAGCAAAAAAAAAAAAAAAAAAAAAAA

>AY837869.1_Tni
GTTATAGTAGCTATTTGGCTTTAAAAAGCTAATACAATGGCTGAAGAAAACTGGAATGACGAAAATGCTG
AGGCGGGCAGCATGGCTGTCGACAGCATGCCTTTACCGCAGGCCGCTGACATTCCCGAAATCAAGCTTTT
CGGGAGATGGAGCTGTTATGATGTTCAGGTGTCTGACATGTCCCTGCAGGACTACATTTCAGTCAAGGAG
AAGTACGCGAAATACTTGCCTCACTCTGCCGGCAGGTATGCTCACAAGCGTTTCCGAAAGGCCCAGTGCC
CCATTGTTGAGCGCTTGACAAACTCTCTGATGATGCACGGACGTAACAACGGCAAGAAACTTATGGCCGT
CCGTATTGTTAAACACGCATTTGAAATCATCCACCTTCTAACTGGCGAGAACCCCCTGCAAGTGCTCGTG
ACAGCTATCATCAACTCTGGACCCCGTGAAGACTCCACTAGGATCGGTCGTGCCGGTACAGTGCGTCGTC
AGGCTGTGGATGTGTCTCCCCTGCGCCGTGTGAACCAGGCTATCTGGTTATTGTGCACTGGTGCCCGAGA
GGCTGCATTCAGGAACATCAAGACCATTGCTGAGTGTGTTGCTGACGAGCTCATCAACGCTGCTAAGGGT
TCATCAAACTCTTACGCCATCAAGAAGAAGGATGAGTTGGAGCGTGTTGCTAAGTCCAACCGTTAAAACT
TTTATTTTAAGATATCTCTCAATAAAAAAAGTCCGAAAAAAAAAAAAAAAAAAAAAAAAAAAAAAAAAAA
AAAAAAAAAAAA

>KM064630.1_Harmigera
TTCGCTCCCGTTGCAATCATGGCGGTGAACAACATTTCTAAAAAGCGAAAATTCGTCGGAGACGGAGTTT
TCAAGGCGGAACTCAATGAGTTCTTGACCAGGGAGCTTGCTGAGGATGGCTACTCCGGCGTGGAGGTGCG
CGTCACTCCGACTCGCTCGGAGATCATCATTATGGCCACCAGGACACAGAGCGTGCTCGGAGAGAAGGGC
CGCAGGATCCGTGAGCTGACCTCCGTGGTGCAGAAGAGGTTCAACATCCCGGAGCAGTCTGTGGAGCTGT
ACGCCGAGAAGGTGGCGACCCGCGGTCTGTGCGCCATCGCGCAGGCTGAGTCGCTCAGATACAAACTCAT
TGGAGGACTTGCTGTACGTCGTGCATGCTACGGTGTCCTCCGTTTCATCATGGAGTCTGGTGCCCGTGGT
TGCGAGGTTGTCGTGTCCGGCAAGCTGCGTGGTCAGCGTGCCAAGTCCATGAAGTTCGTGGACGGCCTCA
TGATCCACTCTGGAGACCCATGCAATGACTACGTCAACACAGCCACACGTCACGTGTTGCTCAGACAAGG
AGTGCTTGGTATTAAGGTCAAGATCATGTTGCCATGGGACCAACAAGGCAAGAACGGCCCCAAGAAGCCA
CAGCCCGACCACATCCTGGTGACGGAGCCCAAGGACGAGCCCGTGCCCCTGGAGCCCACCAGTGACGTGC

GTTCCCTTGCGCCCGCGCCGCTGCCGCAGCCCGTACCCGCTGTCGCTTAGGTCTTAA

>gi|282356537|gb|FE273699.1|FE273699 CF0067861 Spruce Budworm molting fifth-sixth larval instar library Choristoneura fumiferana cDNA clone SH53-A5 5' similar to ribosomal protein S5, mRNA sequence
CTTGTGGCTGACGACAGCGTGGAAGCGGGCAGCATGGCCGTGGACAACCTGCCTCCGCCACCAGCCGCTG
ATATTCCCGAAATCAAACTATTTGGGCGATGGAGCTGCTACGACGTGCAAGTTTCGGACATGTCCCTGCA
GGATTACATCTCCGTGAAGGAGAAATACGCTAAATATTTACCGCACTCCGGTGGCAGGTATGCACACAAG
CGCTTCCGTAAAGCTCAGTGCCCGATTGTGGAGCGTCTAACGAACTCCTTGATGATGCACGGACGCAATA
ACGGCAAGAAGCTGATGGCCGTGAGGATCGTGAAACACGCCTTCGAAATCATTCACTTGCTGACCGGTGA
GAACCCTCTGCAGGTTTTGGTGACGGCTATCATCAACTCTGGGCCGCGCGAGGACTCTACTAGGATCGGT
CGCGCCGGTACCGTGCGTCGCCAGGCCGTGGACGTGTCCCCACTGCGACGCGTGAACCAGGCTATCTGGC
TGCTGTGCACCGGAGCCCGAGAGGCCGCTTTCAGAAATATCAAGACGATTGCAGAGTGCGTCGCCGACGA
GCTCATCAACGCCGCCAAGGGCTCCTCTAACTCATACGCCATCAAGAGAGATGAGCTGGAACGTGTAGCT
AAATCCACCGTATGTTAAGTGTATGGGCTGCCAAATGAGAACGTAGAAA

>gi|54609290|gb|AY769319.1| Bombyx mori ribosomal protein S5 (RpS5) mRNA, complete cds
GGCACGACTTTTGACAGTGCGAGGTTATAATTTCTATTTAGTAATAGAAAAGTACCAATCATGGCCGAGG
AGAACTGGAATGACGACGTAGCCGAGGCAGGCAGCGTGGTTGTGGAAACCATGTCTTTACCACAAGCCGC
CGACATTCCTGAAATCAAGCTTTTCGGCAGATGGAGTTGCTACGATGTGCAAGTCTCTGATATGTCTCTG
CAGGACTACATTTCCGTTAAAGAGAAGTACGCAAAATATTTACCTCATTCAGCTGGCAGGTATGCACACA
AGCGTTTCCGTAAAGCCCAGTGCCCAATCGTGGAGCGCCTTACAAACTCTCTAATGATGCACGGTCGGAA
CAATGGCAAAAAACTGATGGCCGTACGTATTGTCAAACATGCGTTTGAAATTATTCACTTGTTAACTGGA
GAAAACCCTCTGCAAGTACTCGTGACTGCCATTATCAACTCTGGACCCCGTGAAGATTCGACTAGGATCG
GTCGTGCGGGTACAGTTCGTCGTCAAGCCGTTGATGTTTCACCCTTGCGCCGAGTCAACCAAGCAATCTG
GCTTTTGTGCACAGGTGCACGTGAGGCTGCATTCAGAAATATTAAAACAATCGCAGAGTGTGTTGCAGAT
GAATTAATTAATGCAGCTAAGGGTTCATCTAACTCCTACGCCATCAAAAAGAAGGACGAGCTGGAGCGTG
TTGCTAAATCCAACCGTTAAAATATACTATTAAGTCATCTTTACAATAAACAGGAAACTGAAAAAAAAAA
AAAAAAAAAAA

B) Hel-2 sequences used for the estimation of transposon-derived sequence divergence.

>ET217030.1_Onubilalis
ATCCCAACTAATATTATAAATGCGAAAGTAAACGCTTTCCCGCTTAAACCTCGCAACCGATTTTGATGAAATTTGGC
ATAGAGATAGTTTGAGTCCCGGGAAAGAACATAGGATAGTTTTTATCCCGGTTTTTGAAACAGGGACGCGCGCGATA
AAGTTTTTCTGTGACAGACAAAATTCCACGCGGGCGAAGCCGCGGGCGGAAAGCTAGT

>FF373093.1_Tni
ATCTCTACTAATATTATAAATGCGAATGTAAACGCTTTCACGTCTAAACCACTGAACCGATTTTAATGAAATTTGGA
ACAGAGATAGAGTTGACCTTGAGAAAGAACATAGGATATGTTTTTTTTTATCCCGGACTTTTGAGGAGTTATCTTGG
AAACGCGATATAACCGACATCGACGCGAGCGAAGCCGCGGGCGAAAAGCTAGT

>DQ649133.1_Pxylostella
ATCCTAACTAATATTATAAATGCGAAAGTAACACTCTTTCACGCCAAAACTACTGAACGGATTTGAATGATATTTGG
TATACATATGGTCTAGACCCTGGGAAAGAACATAGGCTACTTTTTATCCCGGAATTCCCACGGGAAAACTTTTTAAT
GCGAAGCGAAGCGCGCGGGAACAGCTAGT

>FP340432.1_Harmigera
AACCCTACTCATATTATAAATGCGAAAATAAACGCTTTCACGTTTAAACCACTGAACTGATTTTAATGAAATTTGGT
ACAGAGATGTTTTAATCCCGGACTTTGGGTTCCCTTGGAAACGCGATAAAACCGAATTCGACGCGGGTGAAGCCGCG
GGCGGAAGCTAGTT

>FE274042.1_Cfumiferana
ATCCATACTAATATTATATATGCGAAAGTAACTCTGTCTGTCTGTCTGTCTGTCTGTCTGTCTGTTACGCTTTCACG
TCGAAACCGCTGAACGGATTTTGATGAAATTTTGTATCGAGATAGTTTAAGCCCCAAGGATCAACATAGGATACTTT
TTATTCCGGTAGAGGGCGCCACTGCGTGATAACCTAGGTCCGCGCAGACGAAGTCGCGGGCATAAGCTAGT

>AP009031.1_Bmori
aTCCCTACTAATATATAAATGTGAATGTAAGTTTGTTTGTAACGCTTTCACGCGAAAACTACTCGACCGATCATCAT
GAAACTTTGTACACATATTCTTGGAGGTATTAGAAGTAGCATAGGATACTTTTTATTAAGAAGAAAAAAAATTTTTT
TACAAAAAATTAAAAAGTTGTTTGTCAAAAAATCTCAAAAATCTAGCTTCTTCAACGCCATCTACCGGTTCAGCAAT
GAAGTTCCAATCCTGAGTACTGTAAATTAAGTGGGGGGGGGGTAAAATGTAGCGTTATGCCAAAGTAACTATTCCAC
GCGGACGAAGTCGCGGGCAAAAGCTAGt
