# Supplementary material for: Horizontal transfer of a non-autonomous Helitron among insect and viral genomes
Source: BMC Genomics. 2015 Feb 27;16(1):137. doi: 10.1186/s12864-015-1318-6 (PMC4344730; doi:10.1186/s12864-015-1318-6)
Supplement: Additional file 4: File S3. — GenBank accessions used in Figure 1 of the main text annotated with Hel-2 sequence features of a 5′-TC and 3′-CTAG, and (CTGT)n or (GTTT)n microsatellite repeats are indicated. Flanking genomic DNA is in small caps. [file 12864_2015_1318_MOESM4_ESM.pdf]

Supplementary File 4 | GenBank accessions used in Figure 1 of the main text annotated with Hel-2 sequence features of a 5'-TC and 3'-CTAG, and (CTGT)$_n$ or (GTTT)$_n$ microsatellite repeats are highlighted. Flanking genomic DNA is in small caps.

>JI832733.1|:199-729 TSA: *Copidosoma floridanum* isotig01588.Coflemb mRNA sequence
agtgattgttaaTCCCTACTTCCCTACTAATATTATAAATGCGAAAGTAACGTCTGTCTGGTCTGTTACGCTTTCAC
GTCTAAACCACTGAACTGATTTTAATGAAATTTGGTACAGAGATAGAGTTGACCTTGAGAAAGAACATAGGATAGTT
TTTATCCCGGACTTTTGAAGAGTTCTCTTGGAAACGCGATATAACCGACATCGACGCGGACGAAGCCGCGGGCGAAA
AGCTAGtaataaatatattttttattactaactatttacttacgaaatattacctaaccttaatgtatgtactgttta
atataatatttaaataaaccgcctctagcatacctactttaaaattttaaacggaacttggtaacagttgtcgctcg
ctgtaggtgattatcgcgtaggtataggcgagttacgacggagtcgacggaggcctctagccaatacatggatgggg
tgggagtgaaatatttggaaaaaaaattaatggaaaaatgggatagtgtaaaacatactctttcttatt

> FF369288.1| TN-LN-384-01-libF_I02 *Trichoplusia ni* whole larvae normalized cDNA library Trichoplusia ni cDNA clone TN-LN-384-01-libF_I02 5', mRNA sequence
atgaaaatgtatgtatgtatcacgttatgtcaaaacgtatttttttttctaatacaacttactaagcgcct
aattttccatacacaactttaaacgactgattaaaacgtttaagattaatatttttttttatttatttcctg
ttgtattagtgttgctctacaattattttgtaattttagtctatcgcttggcaacacttagttttaattg
taacgcacatcagaagcaaaactatgacattattgtggcataatatcgtaagttcttttttaacaaaatg
atacaatttttagtttccgacttaatattcaaattatccttactataatatataatggacttaaaatgt
gaaattcatttaatttaggtactttctatatttctttataagttttactatttattataaaacagtcaga
ttaacttggtctattttcaaTCCCTGCTAATATTATAAATGCGAAAGTAACTCTGTCTGTCTGTCTGTTA
CGCTTTCAGGTCTAAACCACTGATCTGATTTTAATGAAATTTGGTACAGAGATAGAGTTGTCCTTGAGAA
AGAACATAGGATAGTTTTTATCCCGGAATTTTGAAAGGTTCTCTTGGAAACGCGATATAACCGACCTCGA
CGCACGCGAAGCCGCGAGCGAAAAGCTAGaaactaaaagacaattgtatcgtttattaaaaaaagcttgt
gattatgccaatattgtatttaaagtttagaaagcatttataattaattaaatgtgacattcctttgggt
gatttttgtggactatgtattatttttacttt

>CsKBV_32_278bp 19092..18815_*Cotesia sesamiae* Kitale bracovirus segment 32, strain Kitale_HF562927.1
cgggccgctaTCCATACTAATTTCATAAATGCGAAGGTATCTCTGTCTGTCTGTCTATCTGTCCGGCTTTCACGCCA
AAACTGCTGAACCGATTGTAATGAAATTTGGTAAACAGATAGTCTAGAGCCTGAGAAAGGACATAGGTTATAAAACG
TCCCGCTAAGACCAATAGGAGCAGAGCAGGAACAAAAAATGTTGCGGAAACGAGAGCAAACGCGGTTGTACTACATA
CTGCTCCCATTATTTTAACAGGCGCTTATTTTATATCAACTGAGCTCATTCGGCTAGAAGGTTACGCGGGTGAAACC
GCGGGGCACAGCTAGtattttcata

>AP009031.1| *Bombyx mori* genomic DNA, chromosome 2, BAC clone:526E16, complete sequence
gttatgatgtcgatgtattcccatcagagcgtgagattaggtgagttgttgacagcttaattttgtcacagagtaat
tttctaataataaTCCCTACTAATATATAAATGTGAATGTAAGTTTGTTTGTTACGCTTTCACGCGAAAACTACTCA
ACCGACCATCATGAAACTTTGTACACATATTGTTGGAGGTATTAGAAGTAACATAGGATACTTTTTAGTATTAAAAA
AATATATATTTTGTACGAAAAATAAAAAAATTGTTTGTCAAAAAATCTCAAAATCTAGCTTCTTCAACGCCATCTAC
CGGTTCAGTAATGAAGTTCCAATCCTGAGTACTGTAAATGAAGTCGCGGGTAAAATTTAGCGTTATGCCAAAGTAAC
TATTCCACGCGAAGTCGCGGGCAAAAGCTAGtatgatataaatcttgaaaaaaatataattacaccagttatacaaa
aaatacatcaaattatttttcagttaaagacaatttcaaaataaatttatttttctttctgactgtaccaattatcc
gattactccttgtagggaccgtcgcacacaccgcattcgtttggtcaactcctatcaatgacaatgttagcctttgt
actggtgggattgatcaccaccatcagcgctggaccatcaaacgactctttcaaggatcagtgtaagtttgtttata
acaataagtgcagtggcccactcatgcttggtctctttcgatcgtaccgtacagtcagactttcaatgacaaaacat
tttttgacgtagtagctattactactggatccccttaactcatccgtagtagtgttatcctttcctaaattacaaa

>JN992291.1| *Spodoptera exigua* rieske iron-sulfur protein of mitochondrial complex III gene, complete cds; nuclear gene for mitochondrial product
atgacttcggtcacaagggctgggcatttggctccttactttaaggcgacctcatcagtggtttccaatggcctgaa
gcctttggttgtggcgcacacgccagctgagaaggtgttggtgcaccctttgccaaaaacctctaccgtggagactc

tgcatggatccctgcctatccagggcctgaaggctagagtaaatggtcgcggtgagctattttccctatgattttgt
gtttaattgaaatatattccgtaaaatacaagaaaaaatcgtgttacataacaccttgtcaattctaggaccaagtc
atgttcgtttcgcgcataccgatattggttaccccgacttcacggcttaccgccgcaaggagacccaggaaccgacc
gcgaagagcgccgaaactgttgatggacgtcaatccttcacctatctcattgctggaggtaagtccttgaaattttc
aaatcatattttgcttaccaaatactgcaactgaatggttatagtatgcatatcgtaacatatacattattaccgga
agacttttgagaatggtaaagtagtgatgaatgtcttatgtaaacaaaacatggattaacatattactataatgttg
taattcttacacagacttggtgtatttatagtatttgacattaaccttcaaatgccaagaataccattgctgggatt
tgagttacaaaagaattgtgcggaaagttaatatacaattacttacttgtgtcaaaatttcttcatatcttatcat
ggaaaatattttatatattcatccttttttatgataacctgaaactgcagctgagtacatgatgcacttttacttc
aacttacagtaaaaaaatataatgtacagactattgagataacaaataaaatatcatctattacatattaggtatta
catttatatgtcataattttcacaaagaagttttaaacacaactttgaacttataattcttagtaatcagatgaagg
gtgctaaaagaaaataggaaaattatagaatattacttaaaatgctgcgattttgctacaacatgaagaaacaaact
aaatgcttataagaatgatctcatttgagtatgttaattaagtttatgttccatctggttttaacaaaaatataaag
tataaaacttgaaaagaaaaacaaaaggttacgcttaaagaattattcagtaaaattagcttcaaataatcggagtt
ctactataatatatttgttctacaactgtaacttaataaaaaaagtttcactgatattctttcaaagtagcaaaatt
gtttataaaaccaaaatttattgaccttcttgggaatttacacactaaatagtatatttctaacaatcctcagctgg
tggtgtggccggtgcgtatgctgccaagtcgatagtgacacactttgtgtcatcgatggctgctgctgctgatgtct
tggctttggctaagattgagatcaagttgtctgaaatccccgagggcaagtctgttaccttcaagtggcgtggtaag
ccactgttcatccgtcacaggtaaggaaattttcatttactgtttttatgctagcattctttttgttaattgaata
ttggatgtagaaacttactacacacttaattaaggaaataaaaacacaatagaaacataataaaaatcatttttttgt
acagaaaaaaacaaactgaaataaatgttcttaaaaaaaaaaaggttgagagagagcaagagagaagagtagttgaaa
gaaaagaaaacaattttcgtatcatatcattcttaaataataaaagacttgccagaaatggtattattatgaaagta
tggtttactattcaatgtttcaattgtagaacaaattgattattacttatggctacagatctatctgtgtatcctaa
taactggtttatacttatatcgggtactcaattacctactcatatttatgaaaacaataaaacaccaaaatattgat
caattttttttttttaattttgcgtttggctggtagttatagtattagggctggatacattcatactctgggcacaat
gccagacaccatggtattattaaaaacttaacctaagtgtgcatataaaaccaatgtaatgaaaattttgtttgcag
tgtcgaaattaatctcaagatctctttattgagcatatgcagataacatgggatagtgttgcccatgctccataaaa
atatcataatcggtctaaatgtaatcaattaaaccgtatcataaagtaatggcttaattgtgttaagtaacacaaag
ctctactacttgcaagtcacaatggactccttattcatgaatagcttgagcatcacggcagcatgtgtagaactttt
ttcagttagattagtaaactgttatctatacttcaatactaataacattattaagaggtaaaatttgtaagtttgta
tgtaggggggttatctttgaaaacgctgctcagattgccaaaattctttgctgatagatagctacaatgttcctgagt
gctataggctataaaacatcacgctactgtaaaggaaccgcgtgagagtagctgttaTCCAAACTAACATTATAAAT
GCGAAAGTAACTCTGTCTGTCTGTCTGTCTTTTCATCACGCCTGAACTACTGAACCGATTTCTGTGATATTTGGTAC
AGACATAGTTTGGAACTCTGGAAAGGAAAAAAATAGTTTTTATACCAAAAATCTGCAGGAAGTCACTATTCCTCGCG
AACGAAGTCGCGGGCAAAAGCTAAttttcaataaattgagtacctacctatgtctcacgatatttattatatcaaaa
tatgtcgtccaaaattgttgttactaaatattatgttacttataggaaatattattatcaaaacagtaatggtacct
acaacatatttatatatttagttttctagaaagtagaatcttaaattagctacgcagactttccattgccaaataat
aagaatattgtttgtcttccctttgttagctactacgcatgagagattcaagtatgcatgacacatgtgattcatct
catagaatacgtatttatctttagtaatacactatacatatgtagtgctgttattgtaggtataggtaatagtttgt
tgggcattaatttaaaaaaactgaaaagattaataataattgattaaaatatttttaggtatatgatacgtttagga
atgtaaatacttataggcctattccatattcaagactgacaaattaaactgcgccaacctatctatatggttggttc
tgtataattattattgtaatatactttgttttatattgaaaactcaatgagacaaagtgttttgtgagttgtactct
cgtttatgagaaataaacaaaataaagttatattaaacatctgggcccacgacatagaatagggatttacattcatt
gtatcattttaaacttgtattatatccgcaaaattaccattccagaaccgaaaacgagatttcaaccgagcaggcag
tgcctgtagacaccctgcgtgaccctcagcacgacaaccagcgcacacagaaccctaagtggctggtagttattggt
gtatgcacccatcttggatgcgtacccgttgccaacgctggagacttcggaggctactactgtccctgccacggatc
ccactacgacgcttctggccgcatccgcaagggacctgcccctcttaacttggaagtccccccacacagcttcgtcg
aagacggcctgctagttgtaggttaa

>gi|14009480|gb|AF254789.1| *Autographa californica* nucleopolyhedrovirus
mutant vsk-1d1, genomic sequence
CCAAACTAATATTATAAATGCGAAAGTAACTCTGTCTGTCTGTCTGTCTTTCTGTCTGTCTGTCTGTCTTTTCTTCA
CGCCTAAACTACTGAACCGATTTGTGTGAAATTTGGTACAGACATAGTTTGAAACTTGAGAAAGGACATAGGATAGT
TTTTATTACAAAAAATAAAAATAAAAAATAAAATTTATTACGGACATACTATATAATAGTGCCATCTATTGGTCAAA
TGTCGAGCTGTTCCTATGCTCCGTAGATAGATGGCGTTAATCGCGCAATGGTGTCATTACACGTGTTCGGTTCATGT
TATTGTTTTAATTCATCGGAAAATCCATCAGAAAAATGTAAATAAACAGTAAAAAGGTGTAAAAAAAATAATATTAA
TATAATAAAAGTTTTACTACAAAGAAAATGCTCTAACGGAGTATAGATAATTCTATTAGTACTACGCGCAATGGTGT

CGATCGTTACACGAGTTCGGTTCATGTTGTTTTAATTCATCGGAAAAAAGTAAATAAATAGTAAAAAGGTATGAAAA
AAATAATAAAATAACATATAAAAAATATAATACTACTTTTAGTACAAAGAAAATGCCCGAACGGAGTATAGATAAAT
AATCCGAGTTGTCACTATGGTCGATAGATGGCGTTGGGATCGAAATAGATCGCGCTATGGTTTCGTTACACGCATTT
GGTTGGGTATCGGCCGTGCGCTTCGGTACCGTCGTGGTAAAATTGTATTGTCGGTTGTATGGTCGTCTATGTGGATC
GGTCCTGTTTCGTAAATTCTAAATTGGTTCCGTTGTTTGATGTAGGTTTGATCTCGAATATTAGTACTTAGTTATTT
TGTTTAGTAAAACTGTAATTCTAAGTTCGTTTTAAATTATTTAGTTTTAAGCTTGGTTGCTTCTCAAAAATCCCGCG
AGATCGGGAACTATGTGAGTAAAACCAAAAATCTGCCGGAAGTCACTGTTCCACGCGAACGAAGTCGCGGGCAAAAG
CTAGt

>HN309366.1|HN309366 Cm34_N01.r *Cucumis melo* BamHI-BAC library (BCM library) Cucumis melo subsp. melo genomic, genomic survey sequence reverse complement
caccgctgcctctgctcttgctacggcagaagaaacggcgcaacaaactctttctcgtcttttagttaacaatataa
tcaaTCCATACTAATATTATAAATGTGAAAGTAACTCTGTCTGTCTGTCTGTCTGTCTATCTGTCTCTTCTTCACGC
CTAAACCACTAAACCGATTTGAATGAAATTTGGTATGTAGGTAGCTTGAACCCCAAGGAAGGACATAGGCTACTATC
CGACTTCGACTTCCTAAGCACGCGCGAGATAACCGACATTCACGCGGACGAAGTCGCGGGCAAAAACTAGtatcaaa
taaggaagtagggacatacacatacttgacacctttattacatttgcagacgtagattgaaaatcgcgactctcata
gccccactaaccgtcccacgcgctgtttaattcaactggtttataactaacaatgtaaatcacaaacagaggacatg
tcgtgcttaatgcactctctgaagggttcaactttaactaaggttcaaaacaactagttaaagctgacagaatactc
ttaaagttacaagacttggttcttagatctgtattaaggacttaagttaagtttaactgtgttgttaatggtttttt
tgagactgggttggtataacgccttcctctcttctacttgctgtgcccannnnggatcc

>HN303792.1|HN303792 Cm60_F04.f *Cucumis melo* BamHI-BAC library (BCM library) Cucumis melo subsp. melo genomic, genomic survey sequence reverse complement
tatctcctcttttcccatcttggaccagtcacaccctcatatttattataaaactacaattcaattgatatcgttca
gccttacttgcctgaactgaaTCCATACTAATATTACAAATGCGAAAGTAACTCTGTCTGTCTGTCTCTTCTTCACG
CCTAAACCACTGAACCGATTTGAATGAAATTTGGTATGTAGGTAGCTTGAAACCCAAGGAAGGACATAGGCTACTAT
TTATCCGACTTCGACTTCCTAATTACGCGCGATATAACCGACATTCACGCGGATGAAGTCGTGGGCAAAAACTAGta
ttctatattcgtcgaacgtatactgagcccgtccaacctgtcgcgggttcgatgcccagtttgaacaaaattgtgtt
tgattaacataaacgccacaatttttttttaaatttaaatctatttttgctcaagcattataaaaaacttttcccta
gagcgggaatggcgtaaatctagactacaataccattgacggcgatgattataatattgtgttacttaatataatct
cacttctcgtataaagtactaatagtaaacagcggcttcaaagaatgacggccagcccgccaagatcccttagtact
ctttggatncc

>HN303759.1|HN303759 Cm60_D03.f Cucumis melo *BamHI-BAC library (BCM library) Cucumis melo subsp. melo genomic, genomic survey sequence reverse* complement
ctcttttcccatcttggaccagtcacaccctcatatttattataaaactacaattcaattgatatcgttcagcctta
cttgcctgaactgaaTCCATACTAATATTACAAATGCGAAAGTAACTCTGTCTGTCTGTCTCTTCTTCACGCCTAAA
CCACTGAACCGATTTGAATGAAATTTGGTATGTAGGTAGCTTGAAACCCAAGGAAGGACATAGGCTACTATTTATCC
GACTTCGACTTCCTAATTACGCGCGATATAACCGACATTCACGCGGATGAAGTCGTGGGCAAAAACTAGtattctat
attcgtcgaacgtatactgagcccgtccaacctgtcgcgggttcgatgcccagtttgaacaaaattgtgtttgatta
acataaacgccacaatttttttttaaatttaaatctatttttgctcaagcattataaaaaacttttccctagagcgg
gaatggcgtaaatctagactacaataccattgacggcgatgattataatattgtgttacttaatataatctcacttc
tcgtataaagtactaatagtaaacagcggcttcaaagaatgacggccagcccgccaagatcccttagtactctttgg
atcc

>gi|364284157|gb|HQ717469.1| *Colias eurytheme* clone PGI4-45_10 phosphoglucose isomerase (PGI) gene, intron 4 reverse complement
ctggggatatatttatttatgtcaataataattaataatcatattatcatattaatattataaatgggaatattaat
aaaacttaaataaggaaaactatttaaataattttataatttatcaataaaatcagtataactattttgttggtcat
atatgtagattaatagatattaTCCATACTAATATTATAAATGCGAAAGTAACTCTGTCTGTCTGTCTGTTACTCAA
TCACGCCTTAACTACTGAACTAATTTGCATGAAATTTGGTATAGAGATATTTTGATACCCGAGAAAGGACATAGGAT
AGGTTTTATCCTGCAAATCCTTCGGGAACAGGTTTTTCCTTGAAAACGCGGGCGAAGCCGCGGGCGGAAAGCTAGta
tctttatataaattatcatcataaataatgacaatgtgtattttattgatgactcatagtatatcatgtgtaggatt
aaaattcttaaagatattaatgattacatac

>FP340420.1gi|291201245:26084-29194 70A06_SfBAC_fin, *Spodoptera frugiperda* BAC, egg DNA reverse complement

aTCCCAACTAATATTATAAATGCGAAAGTAACTCTGTCTGTCTGTCTGTCTGTCTGTTACGCTTTCCCGCTTAAACC
TCGCAACCGATTTTGATAAAATTTGGTACAGACAATCTTTAGACCCTGAGACAGAACATAGGCTACTTTTTATTTCG
AAAAAAAGGGATGAAGGGGTTGAAATAGAGGTTGAAAGTTTGTATGGGATTTCTTAATTTTAAAAGATAAAACCATG
AAACTTTATATTTAAGCACTTGATAAGAAATCATTAAACATTTGTTCTAGCATTTTTGAAATTTCGACCAGCTAAGG
GGTGAAATAAGGGTTGAAAGTTAGTATGCAAGTTCGTCAAATATCACGTCATAAGAAACAAAAAATCGTTTGTTCTA
GCATTTTGAAATTTTTACCAGTTTGGGGGTGAAATAGGGGTTGAATTGAAGATAAGACCATGAATCTTGGTACGTAG
GTCTCAAAGGATGTGCAGAATTTTTCTACAAACGGAATTCCCGAAATTCGAACGGAACGGATTTGGAACTGGAACTA
CAAAGCCAAACTTTTTGTATACGAAATCTTAATCACTGAACCTAGGAACATGTAATTTAGAAGTTTTGGGTGTATCA
AGTTACAAAAAAGTTCGTTTTTGAAGTTTAAATTCCTTTTAACGAATACTCTGCATCGCCGCCGCTGCCGTCGGTCG
TCGTCGCGCACATTCGCCGAAGTGTGGCGGCATCTGCGGCAGGCAACGGCGCGGCGCTGTCGTGTGGTGGGGGAGCG
CACGAATCTAACTATGCCTACCCGAACGGGCAAACACGTGAGGGCAGACGTCGTTGTCGGACGCATGCGATTAGAGA
AATTTGCGCTATAGAGAATTACATTTTTCGATCCAGTAAAATTATTTTTTTTGTTTCGTATAACTTTTTAAGTGTTT
TTTATTAATTATAATTAGGTCCTGTAAATTAATTTTGTACATACTTGATATTTCTTTTAGTTCATAGTTAAAAGTAG
GTAAGATAGTTTCCGTTACTTTAAAAAAATCCGATTTGATTTTATTTCCTTTTTTTAATTAATCTAAGAGGTCGTAG
TAGGTGTAAAACTAACCTAAGCTACTAACCTCCTACTTTTTTATGCATTGGTTGTAAATAATATTGTAATGCTGTCC
AAAACTTGGAAGATCTTAAAAACAACGTCTATCCCGGTTTAGAATATAACATGAGGAATAGAGAATGGCTGTGTGAA
AGGGCAATATTAGCACCGACCAATGAAATCGTGGGAAAGATCAATGAAAGAATGATGTGACATGTGCAAGGCGATGT
TGTCGAATATTTGTCAGTAGATATTGTCATGGACAGTGAACAGGTGACGTCTTATCCTACCGAGTTTCTAAACTCTT
TAGAGCTGTCAGGAGTGCCGTCTCATAAGCTGAGACTGAAAGTTGGGGTTCCAGTTTTATTAATGAGAAATCTCGAA
GCGCCGAGACTATGCAACGGGACTCGATTGCAAATTACGCAGCTAGGACGCAATATTATTGGAGCAATTATAATGAC
AGGTATAGCTAAAGATGAAGAAGTTTTGATTCCACGCATACCCATGATCCCTACAGATTTACCGTTTCAGTTTAAAA
GAATCCAGTTTCCCTTCAAACCAGCCTTCGCAATGACCATTAACAAAGCGCAAGGGCAAACACTTAAAGTAGCAGGT
GTCCATTTAGAGAAAAACTGTTTTTCGCATGGCCAACTGTACGTGGCCTGCTCGCGAGTGTCGAGTCCAAACAATCT
CCACATTTTAACAAATGATAACAAAACAGCAAACGTTGTGTACAAAAATGTTTTAAATTAGGTCATTTAAAAATATT
TTTGTACACAACGTGAGGCTGGCCCCTGTAAGAGTCTCTTAGACATAAAGGACCAGGATTGACAAAAACTAATTTCA
CGCGGGCGAAGCCGCGGGCGGAAGCTAGtggttaatataaacatttttaacagtatgttttaaccattaataacagc
tccatcgaatcctttaagaaatagacgcaagttgtttatttatttattgaattaaaagaagagtattgtacaatata
tggaatatacctactgaaaaaaatattaaacaactgcaaatcttctgaaacaaaaaacctgtcatttatacaatatc
aaaatcagcacaatgtcctacaaaaactggtaatttcagtataaaatcacaatacacctgattacctaaccctaccg
cttatatacatagctatacatatataccagctactaccactacatagtacagtcaccctcgtcccatagtaacaagc
acgagcctcaatcttgaagcacttaactgattgcctgttggacgcgactgtactcgtactcaacaaatgtatagttg
tataccattgtactcagcaaatgtatagttgaactaggtttcttacgcggttttgcacacgtttagtctataaacct
taccaaaaaaaaaattgtgtgaaaattcgttgcgtggttttaaagacttaagattacatagggacagataagtgttt
tttattttattttataccatgtaataatcactacaattttgtttagtcggcacgaatggtccaaactggagtgatac
cacggcctcagagtaaaccggcgtaaaactacgcttactttgtgttgtgtgagactgtgagtgaggttaccagatac
acaatcccctaaaggccggctacgcacttgtaactcctctggtgcgtccatggcctgcgccaattgcttttcatcag
gtgactggtctgaacatttgccaactttctttaaagaaatggtgtgtcattgtactcaagaaataaatggtatacgt
cctcggactagagtatcattgtacttttactgttgatttatttgctaggccgtagttccctctatttgaagtaggct
tggaatgtactagtgattacttaaggtgtattaatcgtagttattgatgaataatgcggcgaggtattgagtaattt
gtaggattatgttattttcacacaaaaaagaaatcatttaatgtgaggatatagctagagataatgagaacaaatc
aaattgttttctttttatcgaacgggagttt

>AB597304.1_*Ostrinia nubilalis* OR gene
gcacactaaccttaaatttattgaaaccaaacttaatgtatgtgtttaggctaagtttttatacagtaataaattta
ttctaaaggacaatgacatcatccattttggtccaaaatcaaaagtgccggccaaaaaaataaaagtgctgtatatt
gacagaatacgtctgccttagcatttcttactatggcaccaagcctgtagcaccactgtgcgtcgagtattcttatt
ctttattctgtgaaccgcattaagattttgatgcaaaaataaaaaaataatcccaaTCCCAACTAATATTATAAATG
CGAAAGTAACTCTGTCTGTCTGTCTGTCTGTCTGTCTGTCTGTCTGTTACGCTTTCCCGCTTAAACCTCGCAACCGA
TTTTGATGAAATTTGGCATAGAGATAGTTTGAGTCCCGGGAAAGAACATAGGATAGTTTTTATCCCGGTTTTTGAAA
CAGGGACGCGCGCGATAAAGTTTTTCTGTGACAGACAAAATTCCACGCGGGCGAAGCCGCGGGCGGAAAGCTAGtaa
taaatattgggggctccccatactaagaactgaaactccaaaaatttttttcatcaaacacatacgtgtggggtat
ttatggataggtcttcaaaaatgatatcgaggtttctaaaacatttttttctaaaccgaatagtttgcgtgacaga
cgcttccaaagtggaaaaaagttggtcccccccctctaacttctaaaataagaaaatgaaaaatctataaaaaacat
aaatgatgtacattactataaaaactaccaacgaaaattgttttgaacgagatctagtaagtagttttttttaatac
ctcgtaaatcgtaaaccgcttattaccgcgtaatctttcatactaataacttaaataaaaaataataatttttattt
cataactcaatggtacggaaccttcggtgcgcgagtccgactcgcacttgtttatgaatatacgaaattaaaatcaa

tgaaagcagagtcagtagactcctgcgtgtctttttgtgaagaagacgttatattgcagtacctacggaataccgtg
gtggtacttcgcctgtagcattatgcttgtaagttaa

>DU602177.1|DU602177 OO__Ba0099L05.f OO__Ba *Oryza officinalis* genomic clone
OO__Ba0099L05 5', genomic survey sequence reverse complement: reverse
complement
ccattcctatgtactgactatgacttataaaaaaatgcatacaattacatatacacttcttactttcttacaatgtt
aatatacatttattcattgtgttagcaaataatgaaattattattgtagcaaaaatatatactattaactgacaca
taccttatcttgtaatagaagttagctattaaccaaatttatgctatagactacataaccaattcctgtctccttaa
aggattcttacataatcttcagctttgcacagaatcacttttaattacacgtttatTCGTTTATTTAACTAATATT
ATAAATGCGAAAGTGACTCTGTCTGTCTGTCTGTCTGTCTGTTACGCTTTCCCGCTTAAGCCTCGCAACCGATTTTG
ATGAAATTTGGTACAGACAATCTTTAGACCCTGAGACAGAACATAGGTTACTTTTTATTTCAAAATAAAAGGGATGA
AGGGGTTGAAATAGAGGATGAAAGTGTGTATGGGATTTCTTAATTTTAAAAGATAAAACCATGAAACTGTATATTTG
AGCACTTGATAAGAAATCATTAAACATTTGTTCTAGCATTTTAGAAATTTCGACCAGCTAGGGGGTGAAATAAGGGT
TGAAAGTTATTATGCAAGTTCGTCAAATATCACGTCATAAGAAACAAAAAATCGTTTGTTCTAGCATTTTGAAATTT
TTACCAATGTGGGGGTGAAATTGAAGATAAGACCATGAATCTCGGTACCTTAGACAAAAGGGACCAGGGTTGACAAA
AACTAATTTCACGCGAGCGAAGCCGCGGGCAGAAAGCTAGtaatataatattttcctagt

>FN544262.1 *Cnaphalocrocis medinalis* mRNA for CYP6AE30 protein
cgacaaattgacgtcatttttaggtggagatagttgaagggattgagagtaacatgaatacttttgcccctttct
aacccctcatttagggaagtgggggcgcgagcgaagccgcgggcaaaagctagtactgtgtaataccttgtttaa
gaattgaaaaatcacaataatgatgtatgttttaataatcctaactTCCTAACTAATATTATAAATGCGAAAGTAAC
TCTGTCTGTCTGTCTGTCTGTCTGTCTGTCTGTCTGTCTGTCTGTTACTCTTTCACGCCAAAACTACTGAACGGATT
TAAATGAAATTTGGTATACAGATGATCTAGACCCTGAGAAAGAACATAGGCTACTTTTTATTGCGGAAAAGGGGGTT
GTAAGGGGTTAAAAGGGGGGTAAAAACTTTTCCACGCGGACGAAGTCGCGGGCGGCCGCTAGttttttttataaaaat
atgtacaatatttaattacaaatacttaatcacaactcagttctttggcgtagcgtaaaataaaataaaaatagctg
gtatacaaattattatcctcaattttggtaaaaaccctagactcccacccttccctgggg

> CW664556.1|CW664556 OG_BBa0018K10.r OG_BBa *Oryza officinalis* genomic clone
OO__Ba0099L05 5', genomic survey sequence
gacaggaaaaacggtgctaatgttcagatcgattagcagatcgtgttcgagtcaatcagacaacggttctgtacatt
ggattggccttatgttacgcgctgggtaTCCATAGTAATATTATAAATGCGAAAGTAACTCTGTCTGTCTGTCTGTC
TGTTACTCTTTCACGCCAAAACTACTGAACGGATTTAAATGAAATTTGGTATACAGATGGTCTAGACCCGAAGAAGG
GTCTGTAAGGGGTTCAAAGGGTTGTAAGGGGTTAAAAGTGGGGTAAAAAGTTACTTTTCCACGCGGACGAAGTCGCG
AGCGGCCGCTAGtatatgaatatatttttcccacaccattccactgtctgtagtcttagttcaattcaattaaatt
tattgagtatcacattcttgcagatgagttctgttggctacaagtacctactcgttcttctttctacattagggact
gtgcccattctaatttattaccgattttaaaatactgcaatacagtatcagtgaatatctacatcctttaaccacg
tcttttgctaacccttctgtaacaaatgtatgctactggttattattagttaccgggatagttcgttacacaactag
tgaaatttcttcaaccgattttcattttagtgtcgtgtatctacacacttaaacccttagatcgtatacagtaaaaa
cacgaacacttgtttgtgtgtatgtatttaagtattaaattttgtgtttcctctattgtctt

> CZ077553.1|CZ077553 OM__Ba0074M15.r OM__Ba *Oryza minuta* genomic clone
OM__Ba0074M15 3', genomic survey sequence
agcagagaattccgccagcaagtgattcatctcgcaaggtcgaccggtgcttaacgccgtaatttacaatttctatt
ttcccttgttaacgtgcgaagaccgtgggttcaattatcagtcccggccgtgtcttcatcgcgggcatgggtcactt
gacctaccaacacatatagttaacattttacagacgtgctggcgagctaggtttccaaaggtatgggttagcatcgc
caagtgttagagcattggtgggatattgagcttctgtagaaacgactatcattgcaatataataaatccatacTCCA
TACTAATATTATAAATGCGAAAGTAACTCTGTCTGTCAGTCTGCTACTCTTTCACGCCTAAACTACTCAACGGATTT
AAGTGAAATGTGGTATACAGATAATCTAGACCCTGAGAAAGGACATAGGCTACTTTTTATTGCGAAAAGGGTGTTGT
AAGAGGTTAAAAGGGGGGAAAAAAGTTACTTTTCCACGCGGACGAAGTCGCGGCCGCTAGtaataaataaattctca
caatctcacaatatctcatacattgacctgactccgtactagtagtagcttatgcacttgtgttacagatatcagac
aacagaggaaacacaaatatactacatacttacatatttacacccca

>HQ009530.1 *Cotesia vestalis* bracovirus segment c7, complete sequence
>Untitled reverse complement
tgaaggctcatttggacatgacgaaacagtcgcaaaatattttgaacccttctacttaaatttaagtgaattaagaa

cgatgatactctgcttatgaaagatatttttgacggagatttagctcacttaaaaacgatgttagaattagcaaatt
tagagttaatacctttgtagatcggttagaatagttaagttgtgtataTCCCTACTAGTATATTAAATGTGAACGTA
GGTTTGTTTGTTACGCTTTCACGCGAAAACTACACTTAGCCGATCATCATGAAATTTTGTAAAAATATTTTTATAGG
TTTTAAAAGAAACATAAGATACTTTGTAGTAAAAAAAAATTCATTTTTTACAAAAGATAAAAAAATTGTTGGTCAAA
AAATTAGAATCACATCGATTCAGTTCTATTGGAATGTAATGATCTTTGTAAAAGCTATTATTAAATAAAAATTATTT
AAGAAACTTATGTTGTATTTTTATGCATACCCAATATATTAGTTATGATCTTTGTTTAACGGAGTAGTATTGTTCAT
AATTATCAGGATAGCTACTTGGGATTTCTTCAAGCCTGATAGAAGTCTGGCTCACGACTATTGATAAGTAGGGTCCA
GTTGTTTAGATGAGCATTTATATGTTTGACCTTAAATTTGAACAAAACAAAACGTTAATTTCCTAGtcctattgata
aatattagcatccagatcccgacaattctagcccaagatttttctggcagattctttcttgataattgttgcagata
attgatcaagcttcttgagtcaccatgttgcaattcttgcaaaagtttagtgccatatattatagctaaatttgtca
ttatagataaatttgggaactggggaaatacaggataaagggagagggggaccttactcagtaggaattttcagta
accgaaaaaataattcggacagcccagaccgggactcgaacccggataatttagttatgcgctataggctctaccag
ttaagctatccgagacattgtccgtaactattattcaattacctactagacttctagatcaaaaatctggtggaatc
ttaactttagaatattacttgaaaatattatctaattctgccatttttctatatactgaatcctgatcagctgtctt
aatcaagaaacgttcaattctttacctatatcatgttcacgtatcttgataacctcaccagcaatcgataggaaaaa
tttatttttatcattgaaaagcgtccc

>gb|GAOP01097167.1|:1-658 TSA: *Pachypsylla venusta* comp113589_c6_seq19
transcribed RNA sequence reverse complement
gtgtagtgtaaTCCATACTAATATTATAAATGCGAAAGTAACTCTGTCTGTCTGTATGTATGTTACGCTTTCACGCT
TAAACTACTGAACCGATTTTGATGAAATTTGGTATGGAGATAGACTAAACTTTAGGAAAGGACATAGGCTACTTTTT
ATCGCGAAAAAAAGGGGGTTGGAACTATAAACGGCCATGCCTCTAAGGCGCCAAGCGAAGCGGGCGTATCCTGCTAG
taatattataattggcaaaagaaacgaaaattgtaacattctaggaaatcaatttatcgtttcaaaaataagtgatt
caattatgattacactgattacaaattctatgtatggtatatacatgaatcatgaatcatttgatagaataaatatt
gtggtgattatatcaagatttatgattcaaatatataatgtcttaatcaaaattcaaacgttcaaggttaaaattaa
caaaactgaatattttttttttttaataataaggtaaaatctagaactaatggaaggtttttttttgtttgctacac
gtgttatgtttgaattggatctaaataaggtaatcaaagatagaagttaagatagtgtctataagatactaagccaa
ctatagtgatcaaggtgatatccatacttccatatccatact

>gi|261857392|emb|FP565803.1| H.numata DNA sequence from clone AEHN-7C9,
complete sequence 104720..107590

acccaggtgagtaaatataaataaaaaaatatatttatagcatatttgtgaaagcttattgcttttagct
ttcgccattttaatgtatgtgtttatatggcaacacagttcttttatgtttttattttaaggctatttat
ttaaattattgattgtaaagcaatgacaatacgataatggaatatgttaattctctaggggttaggatct
gtgtgcctctttttgctatttgtgaaaaatgtatgaagttagcttcttatttattttaaaatgatttttt
attattgtggtcagtcgtggcgaagaggtgtcaaccaattcccactcgaggaaatgaaatatttaatatt
ttcctctctttgctccggtgtcgaaattacattaaccatgtacttattcaattatatcctactactatat
attgacattaatattttaaaataaaatacttgccatgcttcctattattaaataaatcatcttgttaatt
taaataaaaaaaatagttaaatcatatataagtaattcaaacttttaaaatttgccatttgttaaacaat
ttattttttccatataaaattactaccaattattaaagtagacggtaatccagtgtttatatttatata
gaggccgttgtggcgcaatgtcgcagtggctaactgtgaacacgacatgatctctctctctctaagttga
cctattcttatgttccaaacaaagcgcgccgtttagttccgccattcaacacgtattgttttaataacta
aacggtggttaaatcttaaaatatacgggatatgccgtgaaagagatttatatttatatcttttatatga
ggccagccgtggcgtaggggcacaaagtgactctaaaatcgactggttgtgggttcgatccccaatcgaa
ggaattaatcattattgcataatattttcatttcttcgctctgacaaaaagataaaagcccggcgttgag
ttccgccactcaacacgcaatgccttcaaatatttggcctaaactggggaatggagagtctttacactaa
gttccctctgcctaccctgctctatatgagatgaattaattttaattaagatttaatcgtctattacttt
agatggctcgcgacgcagatgtctctctattcgcgcagatcacgatgtcgcgacatttcactatgtgttt
gtggaacagtacgcagttttggatgtctagttcttgtttttttgtcatttttacaggtaacattttatttt
agtcaattattattattatatattagtttttaatatgataattaagttgtttttatacaagttaaataaa
gctttttcttcttagttcggagatcaaactgtaatagtataaaaatatttcattattttagtggttttat
tgaatcgattataaatttggagtttagccttgatcagatgtgttcatgtaaacgttatttttcttatttt
cgtggcaaatttaatgttaatataatttctgagacacgttctaccatgccccctattcctacgcgtagga
ttgaccatggggccttcctcgataaatggaccattcaacacaaaaacatttttttcaattccaaccatgag
agcgcgttcaacaaacaaaccctttaactctataatattagtagggaaagattgattgttaaattgctca
acagattatgatatgattccaagtgctattgaactcttgtatattctgaaattatctataatttgtgctg

aagagtaaattatcctcaggtggtatcagtggcgtcgctgggcccgggcgcgctgagcgcgggcggcgcg
gcgtggtgctcgctaacagcgggcgcgctgtccactgcgctaagtttcgcgccccccgcacgctgcgctgg
tgctgagggccgctactaggccggtgttggagtttaatagacgggtgattttgtatttatatttagatat
aaataattatctctctcaaagggttacatgtagacaattcagggaaacttttgtggtattgtgtgattta
aacaatgttttatttgtttttatgaattttagatataaaaggtcttttatatgtgcgattcaaattatca
accatcatacgtatgttcatatttctttacatatgcgaaccagctttgctatcaatatcattcgtgtttt
ttcagtaagaatatataaaatatctatattaggaatcatatacacaatataggaatcatataagttttca
ttcgtcacaaaactgaaataaatcctacaaTCCATACCTATATTATAAATTCGAAAGTGACTCTCTCTGT
TTGTCTGTCTTTTACGCAATCAGGGCCAAACCGCTAATTTAATTATGTTAAAATACTCATATAGAGATAT
TTGAGGGTCTCGGGATGAACATAGGATACTTTTTTTCCATTATTTGTAATATTATCGATATATACCAATA
TATTAAAATACTTATTAATTTGACATGCAATTTATTCTGCCATGTTAATAACAACCGTAGCTAAGCGACC
GTTGCTAAGCAACGATTTGCAAAGAATGTTGTTGTTAAAAAACGTCACCCAAAATTAGCTTGCTTAACGC
ATCTCTCTATTGAAAACTGAAAATAATTTCATAAATATATTTAATAAATATTCGTATAGTTTAAGCGGGC
GTAGCCGCGTTCGGGCCCACTAGtatatatatatgtaaatacacatactccacatgttcacactgactga
ctgaggtaagacgcccgcacagcgttaaccgctgtacgtagaaacgtgaaatttagacagatgtttattt
tctgataaatggaatctggacacccattaaatgtgaaatagtggctgaaagtttgtatggaaaaatctta

>gi|590307250|gb|GAWC01078982.1| TSA: *Aretaon asperrimus* comp58899_c2_seq3 transcribed RNA sequence
aacattatggcagttctgtaaccaaattggtgatttattagatggcatgaagacaacgtatttttgtaat
tatcaatgttcaacaatgtgttttaaactgcaagaagtgagaaaatatatgcaacttgacaagtgttgct
aatgtaccctacaagaaaatcaaactgattttgtggagattatccttcaatgatgcatttccaagtacca
aataacgttagtgacataaatccagctgaaaatcacatgacagttatcatacacaagacactgtagtgag
ctgtaaacagtaatgtgatatcttaatgagatataaaaccatgtaagccaatgaatctgaagttgaattg
gagcaaggataaggtcatgtttacactcgctgtatttactgtgtacgcagtctatattctcataacaagc
atgtttacatgtactaatggtgcagaccagttttccttaccatgaagtgtgctggaataagaagtattac
aattgcatgttttcattgtcggagcttggcaaccaacaacttgatcagcacatcgaatttgcataaTCCT
TACTAATATATAAATGCGAAAGTAACTCTGTCTGTCTGTCGCGCTTTCACGCCAAAACTACTGAACCGAT
TTAAATGAAATTTGGTACACAGATAGTCTAGAGCCTGAGAAAGGACATAGGCTACTTTTTAATGCGAAAA
AAGGGTTGAAGGGGTTGAAAGGGGGGGATGAAAAGTTGTATGGAAGTATCGTCATTTTTAGAGCTAGAAG
CTTGAAACTTATTTTTTAGGCTGTTGATTCGATATAAATAAATATGACATTCAAAGTTTGTAAAAGTTTT
ACCCTCAAGGGTGTAAAATAACAATAGGGAATAGGGGATGAAAGTTTGTATGGAAATATGTAAGGTTTAT
GTGAATGTTTTATAAGTTCCACGAGGACGAAGTCACGGGCACAGCTAGtacttatataaaaacaaatatc
aaatgaattgtattttgtgtaagtttcttgttattttcctgcacacacaacacataagaaatagaacatt
ttgtgaggatttcttgcactggggaaaacaggtgtatttaattatgaccctacattattttcaggatttt
cattcaattctcggttgttacttgtttctagtggaatgatctgcagcgtttgaaataatgttctgtggtg
tgccactagctttgaatggatggcagttcgctaggaaaagaaccttgttgctgatttcctagccaggtga
cttaagcatggccaactcagttacctaacttggccctaaacttctatcaagagtgatgtgaagatgcagg
cctaccacagtgacttgtataattgttgtgaagtatgtcattagtagttgtaatagtcctttaggctaac
taaagaccattatcttgtttcaagtatgcactacatgattcctgtttttttgcagccatcgagatctctgt
gttgtaaaaccacattgatgtgaacactcacaagatttgtcaattgcccaaaaaattttgcattgcatct
gcaaaccaggtactgtctaacaaggtttctcagtttaattgttcatcaaattgccttccaaattacgttg
taaacaaaaaccccacacacttgcctggtattttgaattgtaggccataattgctggagttgtatggggt
gacgagactagcatttctgctccctgaaccacgagccagtgattgaaaataattctactgaaagtttttg
gtgtgtatacaccagcaaagtaggcatcccatcaatgggcatgttgcaactgactaataactatcatctc
agttgtgttgtgttcccacaccttattttgcagattagtatcgggtgtattcaataagtctaaagttggg
aaaaggtcactatgttactatgatggattcaggcttacgtgagcacaattgaaagccctgctcaaaatat
actttcagagatatattggcattttttataaatctgctacaaaacaaatattcaaacaatgtgcacataca
agagtattgtaagccatgctgacacactttacataattatgtatttgccacgctactgagaaatatattt
tctactttaaacgctgagcttagtattcataacaatataacaaa

>APGK01011582.1_*Dendroctonus ponderosae* Seq01011590, whole genome shotgun sequence
aattaataaaaaatagagcatacaaaatataattaattatttgtttaacaattttacatcaatacaatacattattt
tcttattttgtgatcgttgaatttgttatttattgccataaccaataTCCCTACTTCCCTACTAATATTATAAATGT

AAATGTAGTAGTAGTTTGTTTGTTACGATCTTACGCCGAAACCACTCTACTGATTATCATGAAACTTTATACACATA
TTCTTAGATGTTTTAGAAAGGACATAGAATACTTTATATTGTGTTTATTTTATGTGTTTGTGTTGATAAATTATATC
AATCGCGAAAGGAATTAAACAATCCCTGTTTATTCAATAAATCTCATAAGGATATGTAATGGCGTACGCAGTGCTTC
ATTTTCTCCTTGTCTAAGTAACTTTCCAAAGGGAAAACTAATAAAAACATATATCATAGCCATAAATCATATGCGAA
TGTATCATAGGAATGGGCGCGTTCAGCCGACACAACTGTTGCTTACGGTTTCTTGTTGGGTCAGCTGAACGATGCCT
AATAGACATTGGTTACTGTCAACAGTTGGTCAACATCGGCACCAGAAAAGTTGTATGGTGGAAGTTGGTTGATCGTT
GGTTGCTTCGTGGTTATAACCACAATTCTGCATTACAGAGTATATCTGTTTTGTTGTTATGTTGCATTACAGAGTGA
GATAATGTTCTTTGCACTGTGTATGTATGGTGCTATCACTGCACATAAGTCAGTAACATTTGTCCGAGACATTCTAA
AATGACTTTTAAACTCAGACAATGAATAGTTCCCCACTGTTTGAATCGGAAAATTTTCATTATAAACCGGCTTGTTT
CTATGGGAAACAGGTATCGATGGTGCTGCCAGATTTTCTACAACAATCTAACCAAATAGCATTAAAATTAATATAAT
CTTCAAATAGTTCTTGTAACAAAAAACTGTTTTGCCTGTTTATGATGTTTTTCCCTGATTATCAAAGTATGTTGATT
ACTTTATTCGGTTTATTTACTGTGGCGAACTCTATCCGCCACTTCTCAACTAGCAGCTCCAACCGAAACGCCAGCTA
TGTCTCACCGCATAACATCTGCGGCGTTTCGATTGAAACAGAACTTCCATTCTATGTACTCTATCCGCGGCGTCTAA
GGCAGGGCCTACCTAAGCCTTACTGACGAATCCAATAGGTAGACCCGGGACGAAATGCATGGTGCTTCAACGCGCAC
CATATACTTTTTACATAGCCTAACTTTCTTCAAATTGACATATGGATGTCCTTCAGCAAAAATTTAAACAAAGCCGT
TCAGACGAATTAAATATGTTGGGTATCAACAACAACAACATGCTTATGGTTTGACTCA
