# Supplementary material for: Horizontal transfer of a non-autonomous Helitron among insect and viral genomes
Source: BMC Genomics. 2015 Feb 27;16(1):137. doi: 10.1186/s12864-015-1318-6 (PMC4344730; doi:10.1186/s12864-015-1318-6)
Supplement: Additional file 5: Figure S2. — Full GenBank sequence accessions containing Hel-2 Helitrons integrations in the genomes of A) lepidopteran infecting viruses, and B) parasitoid wasps. Helitron-like 5′-TC and 3′-CTAG, and (CTGT)n or (GTTT)n microsatellite repeats are indicated. Flanking genomic DNA is in small caps. [file 12864_2015_1318_MOESM5_ESM.pdf]

Additional file 3: Figure S2. Full GenBank sequence accessions containing Hel-2 *Helitrons* integrations in the genomes of **A)** lepidopteran infecting viruses, and **B)** parasitoid wasps. *Helitron*-like 5'-TC and 3'-CTAG, and $(CTGT)_n$ or $(GTTT)_n$ microsatellite repeats are highlighted. Flanking genomic DNA is in small caps.

A) Hel-2 *Helitrons* identified from viruses that infect Lepidoptera

```
>AF254789.1| Autographa californica nucleopolyhedrovirus mutant vsk-1d1,
genomic sequence_1006..1
TCCAAACTAATATTATAAATGCGAAAGTAACTCTGTCTGTCTGTCTGTCTTTCTGTCTGTCTGTCTGTCTTTTCTTC
ACGCCTAAACTACTGAACCGATTTGTGTGAAATTTGGTACAGACATAGTTTGAAACTTGAGAAAGGACATAGGATAG
TTTTTATTACAAAAAATAAAAATAAAAAATAAAATTTATTACGGACATACTATATAATAGTGCCATCTATTGGTCAA
ATGTCGAGCTGTTCCTATGCTCCGTAGATAGATGGCGTTAATCGCGCAATGGTGTCATTACACGTGTTCGGTTCATG
TTATTGTTTTAATTCATCGGAAAATCCATCAGAAAAATGTAAATAAACAGTAAAAAGGTGTAAAAAAAATAATATTA
ATATAATAAAAGTTTTACTACAAAGAAAATGCTCTAACGGAGTATAGATAATTCTATTAGTACTACGCGCAATGGTG
TCGATCGTTACACGAGTTCGGTTCATGTTGTTTTAATTCATCGGAAAAAAGTAAATAAATAGTAAAAAGGTATGAAA
AAAATAATAAAATAACATATAAAAAATATAATACTACTTTTAGTACAAAGAAAATGCCCGAACGGAGTATAGATAAA
TAATCCGAGTTGTCACTATGGTCGATAGATGGCGTTGGGATCGAAATAGATCGCGCTATGGTTTCGTTACACGCATT
TGGTTGGGTATCGGCCGTGCGCTTCGGTACCGTCGTGGTAAAATTGTATTGTCGGTTGTATGGTCGTCTATGTGGAT
CGGTCCTGTTTCGTAAATTCTAAATTGGTTCCGTTGTTTGATGTAGGTTTGATCTCGAATATTAGTACTTAGTTATT
TTGTTTAGTAAAACTGTAATTCTAAGTTCGTTTTAAATTATTTAGTTTTAAGCTTGGTTGCTTCTCAAAAATCCCGC
GAGATCGGGAACTATGTGAGTAAAACCAAAAATCTGCCGGAAGTCACTGTTCCACGCGAACGAAGTCGCGGGCAAAA
GCTAGt
```

```
>EF710634.1:3121..2809| CsBV_19C4_278bp  element from 3121..2844_Cotesia
sesamiae Kitale bracovirus clone BAC 19C4, complete sequence_
cgggccgctaTCCATACTAATTTCATAAATGCGAAGGTATCTCTGTCTGTCTGTCTATCTGTCCGGCTTTCACGCCA
AAACTGCTGAACCGATTGTAATGAAATTTGGTAAACAGATAGTCTAGAGCCTGAGAAAGGACATAGGTTATAAAACG
TCCCGCTAAGACCAATAGGAGCAGAGCAGGAACAAAAAATGTTGCGGAAACGAGAGCAAACGCGGTTGTACTACATA
CTGCTCCCATTATTTTAACAGGCGCTTATTTTATATCAACTGAGCTCATTCGGCTAGAAGGTTACGCGGGTGAAACC
GCGGGGCACAGCTAGtattttcata
```

```
>HF562927.1|19092..18780 Cotesia sesamiae Kitale bracovirus segment 32,
strain Kitale.
cgggccgctaTCCATACTAATTTCATAAATGCGAAGGTATCTCTGTCTGTCTGTCTATCTGTCCGGCTTTCACGCCA
AAACTGCTGAACCGATTGTAATGAAATTTGGTAAACAGATAGTCTAGAGCCTGAGAAAGGACATAGGTTATAAAACG
TCCCGCTAAGACCAATAGGAGCAGAGCAGGAACAAAAAATGTTGCGGAAACGAGAGCAAACGCGGTTGTACTACATA
CTGCTCCCATTATTTTAACAGGCGCTTATTTTATATCAACTGAGCTCATTCGGCTAGAAGGTTACGCGGGTGAAACC
GCGGGGCACAGCTAGatttttcata
```

```
>EF710633.1|62883..62571 CsBV_17K19_278bp 62883..62606_Cotesia sesamiae
Kitale bracovirus clone BAC 17K19, complete sequence_
cgggccgctaTCCATACTAATTTCATAAATGCGAAGGTATCTCTGTCTGTCTGTCTATCTGTCCGGCTTTCACGCCA
AAACTGCTGAACCGATTGTAATGAAATTTGGTAAACAGATAGTCTAGAGCCTGAGAAAGGACATAGGTTATAAAACG
TCCCGCTAAGACCAATAGGAGCAGAGCAGGAACAAAAAATGTTGCGGAAACGAGAGCAAACGCGGTTGTACTACATA
CTGCTCCCATTATTTTAACAGGCGCTTATTTTATATCAACTGAGCTCATTCGGCTAGaaggttacgcgggtg
```

>EF701642.1|94850..94520 *Cotesia sesamiae* Mombasa bracovirus clone BAC 14G12, complete sequence
tctgttgaagTCCCATTTGAGAATGACGAGGCAGTCGCAAACTATTTTGAACTCTTCTAGTTAAATTGGTCAGGCCA
GCCAATTTAGAGTTAATATCTTTTATTTACGCTTTCACGCGAAAACTATTTAACCAATCATCATGAAACTTTGTAAA
AATATTTTTATAGGTATTAGAAGTAATATAAAATACTTTTAAGTAAAAAAAAAATTTATTTTTTACAAAACAAAAAA
ATTGTTTGTCAAAAAATTAGAATCACATTAATTCATTTATATTAGAATGTAATGATCTTTAATATTTGTCCAAGGGC
TTCAACGCGAGCGAAGCCGCGGGTAAAAGCTATtattaaataa

>EF710635.1|59104..58778 *Cotesia sesamiae* Kitale bracovirus clone BAC 2004, complete sequence
tctgttgaaaTCCCATTTGAGAATGACGAGGCAGTCGCAAACTATTTTGAACTCTTCTAGTTAAATTGGTCAGACCA
GCCAATTTAGAGTTAATATCTTTTACGCTTTCACGAGAAAACTATTTAACCGATCATCATGAAACTTTGTAAAAATA
TTTTTATAGGTATTAGAAGTAATATAAAATACTTTTAAGTAAAAAAAAAATTTATTTTTTACAAAACAAAAAAATTG
TTTGTCAAAAAATTAGAATCACATTAATTCATTTATATTAGAATGTAATGATCTTTAATATTTGTCCAAGGGCTTCA
ACGCGAGCGAAGCCGCGGGTAAAAGCTATtattaaataa

>HF562907.1|6072..6397 *Cotesia sesamiae* Kitale bracovirus segment 2, strain Kitale
tctgttgaaaTCCCATTTGAGAATGACGAGGCAGTCGCAAACTATTTTGAACTCTTCTAGTTAAATTGGTCAGACCA
GCCAATTTAGAGTTAATATCTTTTACGCTTTCACGAGAAAACTATTTAACCGATCATCATGAAACTTTGTAAAAATA
TTTTTATAGGTATTAGAAGTAATATAAAATACTTTTAAGTAAAAAAAAAATTTATTTTTTACAAAACAAAAAAATTGT
TTGTCAAAAAATTAGAATCACATTAATTCATTTATATTAGAATGTAATGATCTTTAATATTTGTCCAAGGGCTTCAA
CGCGAGCGAAGCCGCGGGTAAAAGCTATtattaaataa

Short versions of
>HF586473.1:229709..229450 *Cotesia congregata* bracovirus proviral locus 2 (PL2).
gagaggaaaaTCCCTACTAATATTATAAATGTGAATGTAAGTTCGTTTGTTACGCTTTCACGCGAAAACTACTTAAC
TGATCATCATGAAACTTTATTCACATAGTCTTGGAAGTATTAGAAGTAATATAGGATACTTTTTATACAAAAAAAAA
AAATTATTTTCTACGAAGTATAAAAAAAAATTGTTTGAAAAGATTCATCTCTATAGTAATGATCTCTTATACTTATTT
AATGGCTTCAATGCGAGCGAAGCCGCGGGTAAAAGCTAGtacttgataa

>FR873487.1:33644..33903 *Cotesia congregata* bracovirus segment 29, complete sequence
gagaggaaaaTCCCTACTAATATTATAAATGTGAATGTAAGTTCGTTTGTTACGCTTTCACGCGAAAACTACTTAAC
TGATCATCATGAAACTTTATTCACATAGTCTTGGAAGTATTAGAAGTAATATAGGATACTTTTTATACAAAAAAAAA
AAATTATTTTCTACGAAGTATAAAAAAAAATTGTTTGAAAAGATTCATCTCTATAGTAATGATCTCTTATACTTATTT
AATGGCTTCAATGCGAGCGAAGCCGCGGGTAAAAGCTAGtacttgataa

>gb|HQ009530.1|:1103-1343 *Cotesia vestalis* bracovirus segment c7, complete sequence
gttgtgtataTCCCTACTAGTATATTAAATGTGAACGTAGGTTTGTTTGTTACGCTTTCACGCGAAAACTACACTTA
GCCGATCATCATGAAATTTTGTAAAAATATTTTTATAGGTTTTAAAAGAAACATAAGATACTTTGTAGTAAAAAAAA
ATTCATTTTTTACAAAAGATAAAAAAAATTGTTGGTCAAAAAATTAGAATCACATCGATTCAGTTCTATTGGAATGTA
ATGATCTTTGTAAAAGCTATtattaaataa

>EF710642.1:94510-96503 *Cotesia sesamiae* Mombasa bracovirus clone BAC 14G12, complete sequence
gttgtgtataTCCCTACTAGTTTTTTAAATGTAAATGTAGGTTTGTTTGTTACGCTTTCACGCGAAAATTACTTAGC
CGATCATCCTGAAACTTTGTACTAATATTTTTATAGATATTAAAAGATAAAATACTTTTTATTAAAAAAAAAAAAAA
ATATTTTTTACACAAAATGAAAAAATTGTTTGCCAAAAAATTAGAATCACATCGATTTAGTTCTATTGATTGTGACG
TATCTTGGGCCCAAGATATGTTTGATAATAAGTATACTACAAGTTGGTAATAATATTAAAATTCAATAGATATGTAA
TTGATTTAGATAAAAAATTGTTATTTATTGATTAAATATAATATCATCAATATACATGTTTTTTGATTAAATTTGGGA
CAGTCTGAGCTGTCCCAATTATTTTTTACCGAAAAAGATTTCCACTGAGTTATTTCCTCTTTCCCTTATCCTTAATT
TCTTCCCTCTCCTAAATTCGTCTTCTGTGATGAATTTGTCATTAAATATGGCTTTTTATTAAATACTTTACACTCTC
ATTGTGTTTATACTCTACTTATTGGACCCTAGCGCATCCATTAATACTTGTATCCGGTTGTCAAGTATGTGACAAAA
ATTTACCCAGATACATGACGAATAAATGTTGCTAGAAATTTCCTCGAAATTGAATTAACACAAGCTTATCAAATACC

TATTTTAAATATAGCATCGGACTTCCCGCTATGGAAATATACTGACATTTGAACCGTAGATATTTCGTATAAATTTA
TTATAGCTGAAAAATCTACGTCATATTCGTCTTCAAACCCTATAAAAAGGCTCACATCTTTGGACGGACGCATTCGA
GATCGGAACGTCGAGGAAAGATGTCATTCAAAGTAGCTGTTGTTTTATTTGCAATGATCGTTGTAACTCATTGTGAG
TATCAATATAATTTTTAGTAATTGTTTTCGAAAAAAAAATAATACCGTACTGCTATTAAATAAATTTTAATTATAAAG
TCTGTTGATTATATTAAAATATTTTTTTCTAGGCTCTCACAATGTACCACCGAGGCGGATTTGGAGAAGAGAAAGAA
GAGAAGCACCTTCTATAGTGATTGGAAATGTGGATAATCAAATGAGAAATAACTTTGTTATCGGTGGAAGCGGATCA
ATTCCACCAGGATCTCAGGTGGTTGGCGGTTCTGTATTAACTCAAACAAACAATGAATACGTAACTATCTCAGAGAC
GGGAAAACTGGTAGTGGTAATGCCTGACGTCATTCTCCAGCCAGAACCACTTACTGTTCCTGGATTGAATGACGCAG
AAGTTAGACAACTGCTTAATCTGACTCCGGCTCAAAAAAATGGAATGATAATTATTAATTTAAGACAATCATTAAGT
ATATTAGGTGAATATTGTGAAAGAATTGCACAAAAGGTCGATAAAGCTATCGTTGAGAAGACAGGAAATTTGGAAAA
TGCCAGTGAATCTTCCTACCGTAATCATATTGACGAAAACATGTGGTACATTGAATTAAAAAGTTACTTGGATGAAG
TTTCCAGTAATCAATCTGACCTGGATGAGTTGATAAACAAAATTAAAGTTGGAGATACCATTTCTGATTTGAAAGTT
TCGAGAGCAAGAAGACTGATGAAGAATCTGTTGAAGTCCCATTTGAGAATGACGAGGCAGTCGCAAACTATTTTGAA
CTCTTCTAGTTAAATTGGTCAGGCCAGCCAATTTAGAGTTAATATCTTTTATTTACGCTTTCACGCGAAAACTATTT
AACCAATCATCATGAAACTTTGTAAAAATATTTTTATAGGTATTAGAAGTAATATAAAATACTTTTAAGTAAAAAAA
AAATTTATTTTTTACAAAACAAAAAAAATTGTTTGTCAAAAAATTAGAATCACATTAATTCATTTATATTAGAATGTA
ATGATCTTTAATATTTGTCCAAGGGCTTCAACGCGAGCGAAGCCGCGGGTAAAAGCTATtattaaataa

>HF562907.1:4421..6407 *Cotesia sesamiae* Kitale bracovirus segment 2, strain Kitale
gttgtgtataTCCCTACTAGTTTTTTAAATGTAAATGTAGGTTTGTTTGTTACGCTTTCACGCGAAAATTACTTAGC
CGATCATCCTGAAACTTTGTACTAATATTTTTATAGATATTAGAAGATAAGATACTTTTGTTAAAAAAAAAAAAAAT
ATTTTTTACACAAAATGAAAAAATTGTTTGCCAAAAAATTAGAATCACATCGATTTAGTTCTATTGATTGTGACGTA
TCTTGGGCCCAAGATATGTTTGATAATAAGTATACTACAAGTTGGTAATAATATTAAAATTCAATAGATATGTAATT
GATTTAGATAAAAAATTGTTATTTATTGATTAAATATAATATCATCAATATACATGTTTTTTGATTAAATTTGGGACA
GTCTGAGCTGTCCCAATTATTTTTTACCGAAAAAGATTTCCACTGAGTTATTTCCTCTTTCCCTTATCCTTAATTTC
TTCCCTCTCCTAAATTCGTCTTCTGTGATGAATTTGTCATTAAATATGGCTTTTTATTAAATACTTTACACTCTCAT
TGTGTTTATACTCTACTTATTGGACCCTAGCGCATCCATTAATACTTGTATCCGGTTGTCAAGTATGTGACAAAAAT
TTACCCAGATACATGACGAATAAATGTTGCTAGAAATTTCCTCGAAATTGAATTAACACAAGCTTATCAAATACCTA
TTTTAAATATAGCATCGGACTTCCCGCTATGGAAATATACTGACATTTGAACCGTCGATATTTCGTATAAATTTATT
ATAGCTGAAAAATCTACGTCATATTCGTCTTCAAACCCTATAAAAAGGCTCACATCTTTGGACGGACGCATTCGAGA
TCGGAACGTCGAGGAAAGATGTCATTCAAAGTAGCTGTTGTTTTATTTGCAATGATCGTTGTAACTCATTGTGAGTA
TCAATATAATTTTTAGTAATTGTTTTCGAAAAAAAAATAATACCGTACTGCTATTAAATAAATTTTAATTATAAAGTC
TGTTGATTATATTAAAATATTTTTTTCTAGGCTCTCACAATGTACCACCGAGGCGGATTTGGAGAAGAGAAAGAAGA
GAAGCACCTTCTATAGTGATTGGAAATGTGGATAATCAAATGAGAAATAACTTTGTTATCGGTGGAAGCGGATCAAT
TCCACCAGGATCTCAAGTCGTTGGCGGTTCTGTATTAACTCAAACAAACAATGAATACGTAACTATCTCAGAGACGG
GAAAACTGGTAGTGGTAATGCCTGACGTCATTCTCCAGCCAGAACCACTTACTGTTCCTGGATTGAATGACGCAGAA
GTTAGACAACTGCTTAATCTGACTCCGGCTCAAAAAAATGGAATGATAATTATTAATTTAAGACAATCATTAAGTAT
ATTAGGTGAATATTGTGAAAGAATTGCACAAAAGGTCGATAAAGCTATCGTTGAGAAGACAGGAAATTTGGAAAATG
CCAGTGAATCTTCCTACCGTAATCATATTGACGAAAACATGTGGTACATTGAATTAAAAAGTTACTTGGATGAAGTT
TCCAGTAATCAATCTGACCTGGATGAGTTGATAAACAAAATTAAAGTTGGAGATACCATTTCTGATTCGGAAGTTTC
GAGAGCAAGAAGACTGATGAAGAATCTGTTGAAATCCCATTTGAGAATGACGAGGCAGTCGCAAACTATTTTGAACT
CTTCTAGTTAAATTGGTCAGACCAGCCAATTTAGAGTTAATATCTTTTACGCTTTCACGAGAAAACTATTTAACCGA
TCATCATGAAACTTTGTAAAAATATTTTTATAGGTATTAGAAGTAATATAAAATACTTTTAAGTAAAAAAAAATTTA
TTTTTTACAAAACAAAAAAAATTGTTTGTCAAAAAATTAGAATCACATTAATTCATTTATATTAGAATGTAATGATCT
TTAATATTTGTCCAAGGGCTTCAACGCGAGCGAAGCCGCGGGTAAAAGCTATtattaaataa

>EF710635.1| *Cotesia sesamiae* Kitale bracovirus clone BAC 2004, complete sequence_60747..58778
agttgtgataTCCCTACTAGTTTTTTAAATGTAAATGTAGGTTTGTTTGTTACGCTTTCACGCGAAAATTACTTAGC
CGATCATCCTGAAACTTTGTACTAATATTTTTATAGATATTAGAAGATAAGATACTTTTTGTTAAAAAAAAAAAAAA
ATATTTTTTACACAAAATGAAAAAATTGTTTGCCAAAAAATTAGAATCACATCGATTTAGTTCTATTGATTGTGACG
TATCTTGGGCCCAAGATATGTTTGATAATAAGTATACTACAAGTTGGTAATAATATTAAAATTCAATAGATATGTAA
TTGATTTAGATAAAAAATTGTTATTTATTGATTAAATATAATATCATCAATATACATGTTTTTTGATTAAATTTGGGA
CAGTCTGAGCTGTCCCAATTATTTTTTACCGAAAAAGATTTCCACTGAGTTATTTCCTCTTTCCCTTATCCTTAATT
TCTTCCCTCTCCTAAATTCGTCTTCTGTGATGAATTTGTCATTAAATATGGCTTTTTATTAAATACTTTACACTCTC
ATTGTGTTTATACTCTACTTATTGGACCCTAGCGCATCCATTAATACTTGTATCCGGTTGTCAAGTATGTGACAAAA

ATTTACCCAGATACATGACGAATAAATGTTGCTAGAAATTTCCTCGAAATTGAATTAACACAAGCTTATCAAATACC
TATTTTAAATATAGCATCGGACTTCCCGCTATGGAAATATACTGACATTTGAACCGTCGATATTTCGTATAAATTTA
TTATAGCTGAAAAATCTACGTCATATTCGTCTTCAAACCCTATAAAAAGGCTCACATCTTTGGACGGACGCATTCGA
GATCGGAACGTCGAGGAAAGATGTCATTCAAAGTAGCTGTTGTTTTATTTGCAATGATCGTTGTAACTCATTGTGAG
TATCAATATAATTTTTAGTAATTGTTTTCGAAAAAAAAATAATACCGTACTGCTATTAAATAAATTTTAATTATAAAG
TCTGTTGATTATATTAAAATATTTTTTTCTAGGCTCTCACAATGTACCACCGAGGCGGATTTGGAGAAGAGAAAGAA
GAGAAGCACCTTCTATAGTGATTGGAAATGTGGATAATCAAATGAGAAATAACTTTGTTATCGGTGGAAGCGGATCA
ATTCCACCAGGATCTCAAGTCGTTGGCGGTTCTGTATTAACTCAAACAAACAATGAATACGTAACTATCTCAGAGAC
GGGAAAACTGGTAGTGGTAATGCCTGACGTCATTCTCCAGCCAGAACCACTTACTGTTCCTGGATTGAATGACGCAG
AAGTTAGACAACTGCTTAATCTGACTCCGGCTCAAAAAAATGGAATGATAATTATTAATTTAAGACAATCATTAAGT
ATATTAGGTGAATATTGTGAAAGAATTGCACAAAAGGTCGATAAAGCTATCGTTGAGAAGACAGGAAATTTGGAAAA
TGCCAGTGAATCTTCCTACCGTAATCATATTGACGAAAACATGTGGTACATTGAATTAAAAAGTTACTTGGATGAAG
TTTCCAGTAATCAATCTGACCTGGATGAGTTGATAAACAAAATTAAAGTTGGAGATACCATTTCTGATTCGGAAGTT
TCGAGAGCAAGAAGACTGATGAAGAATCTGTTGAAATCCCATTTGAGAATGACGAGGCAGTCGCAAACTATTTTGAA
CTCTTCTAGTTAAATTGGTCAGACCAGCCAATTTAGAGTTAATATCTTTTACGCTTTCACGAGAAAACTATTTAACC
GATCATCATGAAACTTTGTAAAAATATTTTTATAGGTATTAGAAGTAATATAAAATACTTTTAAGTAAAAAAAAAT
TTATTTTTTACAAAACAAAAAAATTGTTTGTCAAAAAATTAGAATCACATTAATTCATTTATATTAGAATGTAATGA
TCTTTAATATTTGTCCAAGGGCTTCAACGCGAGCGAAGCCGCGGGTAAAAGCTATtattaaataa

>HQ009530.1:15336..16398 *Cotesia vestalis* bracovirus segment c7, complete sequence
ttgtgtacatTCCTACTAGTATCTTAAATATGAATGTAGGTTTGTTTGTTACGCTTTAACGCGAAAATTACTTAGCC
AATCATCCTGAAACTTTGGACAAATATTTGTTTAGGTTTTAGAAGTAACATAAGACACTTTTTATTAAAAATAAAGA
AATTATTTTTTACAAGAAATGTTAAAATTGTTTGTCAAAAAATTAGAATCACATCGATTTAGTTCTATTGGGTGTGA
CGTGTTTTCGGCCAAAGATATGTTTGAAAAATTATATTAATTTAATCTTTGTAATATTTTGGTAAATAATACATCGT
TTGATAATAAACATACCACATGTTCGTAATAATATTAAAATTCAGTAGATTTATAATTGATTTAGATAAAAAATCGTT
GTGATTTATTGATTAAATTTAATATCATCAATACCCATGTTTTTTGATTAAATTAGAGTAAATACGCTGTAGAATTG
AAAAACCCCAGCTAAATATTTATAGGATATGTTGGCTTATTTTAAGATGAACACACACAAAAAACCATGATAAAAGG
GCACAATACGATAACAAGCTTGTGTTGGTCTAACGTCAAATAACCACAACCACAAACCATGATAAAAGGGCACTACA
CGATAACTAGCACATGTTTTGGGTTAATGTGTTTGTTTAAATATATATGTTTTTTGATTTAATTGTAGTAAATACGC
TGTAGGATTCTTAGTGATGATTTATGGTTTATCTTATAAACTAAAACCTAACTTAATGTATGAACCACTGGAAAAAA
AAGTTTGTCTTGTCGCAATACGTTTAAATGTATAGCTTATACAGTTGACGCATTTAAAATGGTTATAAGCTCTACGC
TTAATCGTATTGCGATAAGACTTATACTTTTTTGTCAGTGAGGACAAATTATCCGGTATTAATTAATATTGTATCTA
TTTTCATGCGGATTAATTACCGTGAGAATAATTTGTCGGGGATGAATTGTATTCGGCCCAAAAATCAGTAAACCGTT
CTATTGGAATGTAATGATCTTTGATATTTATCTAAGGGCTTTAACTCGAGCGAAGCCGCGGGTAGAAACTAGttata
tatat

>EF067329.1b|15410..16472 *Cotesia plutellae* polydnavirus segment S41, complete sequence
ttgtatacatTCCTACTAGTATCTTAAGTATGAATGTAGGTTTGTTTGTTGCGCTTTAACGCAAAAATTACTTAGCC
AATCATCCTGAAACTTTGGACAAATATTTGTATAGGTTTTAGAAGTAACATAAGACACTTTTTATTAAAAATAAAGA
AATTATTTTTTACAAGAAATGTTTAAATTGTTTGTCAAAAAATTAGAATCACATCGATTTAGTTCTATTGGGTGTGA
CGTGTGTTCGGCCAAAGATATGTTTGAAAAATTATATTAATTTAATCTTTGTAATATTTTGGTAAATAATACATCGT
TTGATAATAAACATACCACATGTTCGTAATAATATTAAAATTCAGTAGATTTATAATTGATTTAGATAAAAAATCGTT
GTGATTTATTGATTAAATTTAATATCATCAATACCCATGTTTTTTGATTAAATTAGAGTAAATACGCTGTAGAATTG
AAAAACCCCAGCTAAATATTTATAGGATATGTTGGCTTATTTTAAGATGAACACACACAAAAAACCATGATAAAAGG
GCACAATACGATAACAAGCTTGTGTTGGTCTAACGTCAAATAACCACAACCACAAACCATGATAAAAGGGCACTACA
CGATAACTAGCACATGTTTTGGGTTAATGTGTTTGTTTAAATATATATGTTTTTTGATTTAATTGTAGTAAATACGC
TGTAAGATTCTTAGTGATGATTTATGGTTTATCTTATAAACTAAAACCTAACTTAATGTATGAACCACTGGAAAAAA
AAGTTTGTCTTGTCGCAATACGTTTAAATGTATAGCTTATACAGTTGACGCATTTAAAATGGTTATAAGCTCTACGC
TTAATCGTATTGCGATAAGACTTATACTTTTTTGTCAGTGAGGACAAATTATCCGGTATTAATTAATATTGTATCTA
TTTTCATGCGGATTAATTACCGTGAGAATAATTTGTCGGGGATGAATTGTATTCGGCCCAAAAATCAGTAAACCGTT
CTATTGGAATGTAATGATCTTTGATATTTATCTAAGGGCTTTAACTCGAGCGAAGCCGCGGGTAGAAACTAGttata
tatat

B) Hel-2 *Helitrons* identified hymenopteran wasps (Hymenoptera: Ichneumonidae and Braconidae) that are parasitoids lepidoptera larvae

* Note: the sequence flanking all Hel-2 *Helitrons* from *Cotesia vestalis* TSA accessions show 100% sequence identity with those from polydnavirus (PDV) genome segments, which indicate that these accessions are viral derived.

>gi|511508862|gb|GAKG01008729.1| TSA: *Cotesia vestalis* comp1708_c0_seq1
transcribed RNA sequence
tggcttaaacgacacagaagagcggataactcgtcaagacatgggatagtcatgttcaacaatatactgatagctct
gttgctcgtcgcaactatcgacataatcttatgctttggcaatgagtggagtttcaaggtcagccagtgaaatatta
aaatgggccgagtcagaacctagccgtgatttatggcttaaacgacacagaagagcaattgatgaacagtcgaagtg
ggtaaattttgggtcactcaatcggcagtaccaaaataattactactttgatcaaaactcggaaaatggcgatgggt
atcgaaactcgtcggttattacaggatccataacgaatcaggacaaaaattcttacaacagtattggtcaggccact
aacgtatacgttgttctgcctcattggtataatgtttcgaatcagcagacaccctccgccgttcacagaagtttagg
aacaagtatttcacagcagagacgtcaagtagtagaagaaaaaatagagcgattaaggcaaattttggcagcaatac
ataacggagtaaaacaaatagaaagcaggaatcatcgaggcttagaccaaatatttcatgagactactgaagaggaa
tcgaatatgtcattaaaactaaatactttataccgcaagtgtggctcgttagaggcaagacttgactaccaagaact
gcgtcttagacattttaacgatgagagtttgccaatgcaagacattttcgacggagatttagctcatttaaaaacga
tgttagaattagctaatttggagttgatatctttacaggccaattaaaatagttaagttgtgtacatTCCTACTAGT
ATCTTAAATATAAATGTAGGTTTGTTTGTTACGCTTTAACGCGAAAATTACTTAGCCAATCATCCTGAAACTTTGGA
CAAATATTTGTTTAGGTTTTAGAAGTAACATAAGACACTTTTTATTAAAAATAAAGAAATTATTTTTTACAAGAAAT
GTTAAAATTGTTTGTCAAAAAATTAGAATCACATCGATTTAGTTCTATTGAGTGTGACGTGTTTTCGGCCAAAGATA
TGTTTGAAAAATTATATTAATTTAATCTTTGTAATATTT

>gi|511508861|gb|GAKG01008730.1| TSA: *Cotesia vestalis* comp1708_c0_seq2
transcribed RNA sequence
tggcttaaacgacacagaagagcggataactcgtcaagacatgggatagtcatgttcaacaatatactgatagctct
gttgctcgtcgcaactatcgacataatctgggccgagtcagaacctagccgtgatttatggcttaaacgacacagaa
gagcaattgatgaacagtcgaagtgggtaaattttgggtcactcaatcggcagtaccaaaataattactactttgat
caaaactcggaaaatggcgatgggtatcgaaactcgtcggttattacaggatccataacgaatcaggacaaaaattc
ttacaacagtattggtcaggccactaacgtatacgttgttctgcctcattggtataatgtttcgaatcagcagacac
cctccgccgttcacagaagtttaggaacaagtatttcacagcagagacgtcaagtagtagaagaaaaaatagagcga
ttaaggcaaattttggcagcaatacataacggagtaaaacaaatagaaagcaggaatcatcgaggcttagaccaaat
atttcatgagactactgaagaggaatcgaatatgtcattaaaactaaatactttataccgcaagtgtggctcgttag
aggcaagacttgactaccaagaactgcgtcttagacattttaacgatgagagtttgccaatgcaagacattttcgac
ggagatttagctcatttaaaaacgatgttagaattagctaatttggagttgatatctttacaggccaattaaaatag
ttaagttgtgtacatTCCTACTAGTATCTTAAATATAAATGTAGGTTTGTTTGTTACGCTTTAACGCGAAAATTACT
TAGCCAATCATCCTGAAACTTTGGACAAATATTTGTTTAGGTTTTAGAAGTAACATAAGACACTTTTTATTAAAAAT
AAAGAAATTATTTTTTACAAGAAATGTTAAAATTGTTTGTCAAAAAATTAGAATCACATCGATTTAGTTCTATTGAG
TGTGACGTGTTTTCGGCCAAAGATATGTTTGAAAAATTATATTAATTTAATCTTTGTAATATTT

>gi|511508860|gb|GAKG01008731.1| TSA: *Cotesia vestalis* comp1708_c0_seq3
transcribed RNA sequence
tgtaatgaatttccggtctaaaaaaaattaatcagcacatggtaagatctttcttagtaaactactaattaaaatga
tttttttttatcttcacaggggccgagtcagaacctagccgtgatttatggcttaaacgacacagaagagcaattg
atgaacagtcgaagtgggtaaattttgggtcactcaatcggcagtaccaaaataattactactttgatcaaaactcg
gaaaatggcgatgggtatcgaaactcgtcggttattacaggatccataacgaatcaggacaaaaattcttacaacag
tattggtcaggccactaacgtatacgttgttctgcctcattggtataatgtttcgaatcagcagacaccctccgccg
ttcacagaagtttaggaacaagtatttcacagcagagacgtcaagtagtagaagaaaaaatagagcgattaaggcaa
atttggcagcaatacataacggagtaaaacaaatagaaagcaggaatcatcgaggcttagaccaaatatttcatga

gactactgaagaggaatcgaatatgtcattaaaactaaatactttataccgcaagtgtggctcgttagaggcaagac
ttgactaccaagaactgcgtcttagacattttaacgatgagagtttgccaatgcaagacattttcgacggagattta
gctcatttaaaaacgatgttagaattagctaatttggagttgatatctttacaggccaattaaaatagttaagttgt
gtacatTCCTACTAGTATCTTAAATATAAATGTAGGTTTGTTTGTTACGCTTTAACGCGAAAATTACTTAGCCAATC
ATCCTGAAACTTTGGACAAATATTTGTTTAGGTTTTAGAAGTAACATAAGACACTTTTTATTAAAAATAAAGAAATT
ATTTTTTACAAGAAATGTTAAAATTGTTTGTCAAAAAATTAGAATCACATCGATTTAGTTCTATTGAGTGTGACGTG
TTTTCGGCCAAAGATATGTTTGAAAAATTATATTAATTTAATCTTTGTAATATTT

>gi|511488319|gb|GAKG01029272.1| TSA: *Cotesia vestalis* comp9448_c0_seq1 transcribed RNA sequence
agttgatgtgacgttttcattgttttcgctttttgttgatatcgcaaccacttcggcaacggttaccatggcaacag
ttctcatagcaacagtaacaattgtaacggttatcatggcaacggttaaatatgaatgaataaaatttgtaaaagta
ttgagtaaataaattgtcgataataacaattttcacagaagcacgctcgatttttcagtattttcataaattctatc
tcgagtaatttgaaatcctgaccacgttgttaattatttttgtagcgtttgtttagagcatattttcaaaattttt
tccacaaaatattcataaaatttcgtgttcgaggatatttttgttttgaatcctctcgctttttttttgtttgtcttg
tgaaactaacgccgcctatgttttctattctcaatttatctttagtatctactagtttcatactacatttgcagtgt
ttctaaatgtcatcatcttgatTCCCTACTAATATTATAAATGTGAACGTAAGTTTGTTTGTTACGCTTTCACACGA
AAACTACTTAACTGATCATCATGAATCTTTGTACACATATTCTTTGAGGTATTAAAAGTA

>gb|JI832733.1|:199-729 TSA: *Copidosoma floridanum* isotig01588.Coflemb mRNA sequence Hymenoptera; Apocrita; Chalcidoidea; Encyrtidae; Encyrtinae; Copidosoma.
agtgattgttaaTCCCTACTTCCCTACTAATATTATAAATGCGAAAGTAACGTCTGTCTGGTCTGTTACGCTTTCAC
GTCTAAACCACTGAACTGATTTTAATGAAATTTGGTACAGAGATAGAGTTGACCTTGAGAAAGAACATAGGATAGTT
TTTATCCCGGACTTTTGAAGAGTTCTCTTGGAAACGCGATATAACCGACATCGACGCGGACGAAGCCGCGGGCGAAA
AGCTAGtaataaatatatttttattactaactatttacttacgaaatattacctaaccttaatgtatgtactgttta
atataatatttaaataaaccgcctctagcatacctactttaaaattttaaacggaacttggtaacagttgtcgctcg
ctgtaggtgattatcgcgtaggtataggcgagttacgacggagtcgacggaggcctctagccaatacatggatgggg
tgggagtgaaatatttggaaaaaaaattaatggaaaaatgggatagtgtaaaacatactctttcttatt
